# Deep learning to predict emergency department revisit using static and dynamic features (Deep Revisit): development and validation study

**DOI:** 10.1186/s13040-025-00509-x

**Published:** 2025-12-20

**Authors:** Su-Yin Hsu, Jhe-Yi Jhu, Jun-Wan Gao, Chien-Hua Huang, Chu-Lin Tsai, Li-Chen Fu

**Affiliations:** 1 Department of Computer Science and Information Engineering, National Taiwan University, CSIE Der Tian Hall No. 1, Sec. 4, Roosevelt Road, Taipei, 106319 Taiwan; 2 Department of Emergency Medicine, National Taiwan University Hospital and National Taiwan University College of Medicine, 7 Zhongshan S. Rd, Taipei, 100 Taiwan

**Keywords:** Time series data, Emergency department revisit, Deep learning, Hybrid model

## Abstract

**Background:**

Emergency Department (ED) revisits represent a critical issue in emergency medicine. Identifying high-risk revisit cases (revisits with intensive care unit admissions, cardiac arrest, or requiring emergency surgery) is particularly important. While prior studies have explored machine learning models for ED revisit prediction, few deep learning approaches exist, and dynamic features remain underutilized.

**Methods:**

We used data from National Taiwan University Hospital (NTUH), incorporating both static (e.g., age, sex, triage) and dynamic (vital signs) features. A preprocessing strategy was developed to handle temporal irregularities. We proposed a hybrid deep learning model combining Temporal Convolutional Network (TCN) and feature tokenizer (FT)-Transformer to integrate static and short-term dynamic information.

**Results:**

We evaluated our model on NTUH 2016–2019 data, achieving the area under the receiver operating characteristic curve (AUROC) of 0.8453 and the area under precision recall curve (AUPRC) of 0.0935 for high-risk revisits (base rate = 0.01), and AUROC of 0.7250 and AUPRC of 0.2005 for general revisits (base rate = 0.042). The model maintained robust performance when validated on 2020–2022 data. Compared to the static-only logistic regression baseline, our model improved AUPRC from 0.0288 to 0.0935 and precision from 0.0281 to 0.0428.

**Conclusion:**

Our model significantly outperformed the static-only baseline. It demonstrates the effectiveness of multimodal clinical data fusion in improving ED revisit prediction and supporting clinical decision-making.

## Background

In recent years, emergency department (ED) utilization has grown markedly, with the total number of visits in Taiwan reaching nearly 8 million in 2022  [1]. This escalating demand has resulted in significant ED overcrowding, a persistent and globally recognized challenge in modern healthcare systems. Among the many concerns associated with ED crowding, unplanned return visits represent a critical issue and additional burden on the already stressed healthcare system. It is estimated that approximately 5–10% of patients return to the ED within three days of their initial encounter, highlighting the need for more effective risk assessment. In recent years, Taiwan’s healthcare system is heavily reliant on tertiary medical centers for service delivery, resulting in substantial pressure on emergency departments nationwide.

Under such circumstances, ED physicians are often required to expedite the discharge process to free up beds for incoming emergency cases. One critical consequence of ED overcrowding is the risk of unsafe discharges, which may result in unplanned return visits to the ED. Return visits may result from factors such as premature discharge at the initial encounter, diagnostic oversight, or ineffective treatment. These return visits not only place a significant burden on healthcare systems but also cause the increase of medical costs, including resources utilization and the associated national expenditure on health care.

While unscheduled ED return visits are frequently used as indicators of patient safety and emergency care quality, their etiologies are inherently complex and influenced by multiple interacting factors. Some severe conditions may initially present with subtle or non-specific symptoms, leading to missed or delayed diagnoses and premature discharges, which in turn result in early return visits. Notably, a subset of these return visits involves high-risk clinical events, such as admissions to the intensive care unit (ICU), emergency surgical interventions, cardiac catheterizations, or in-hospital cardiac arrest (IHCA) during the return visit. Patients in high-risk tend to experience higher rates of morbidity and mortality compared to the general ED population. A few studies have investigated the contributing factors to such high-risk revisits and emphasized that analyzing their causes can help establish more precise and reliable clinical indicators, ultimately enhancing care quality by enabling early identification and prevention of catastrophic events  [2].

High-risk ED revisits are relatively rare events, necessitating large sample sizes for meaningful investigation. The growing adoption of electronic health records (EHRs) has greatly facilitated research on such infrequent but clinically significant outcomes through the utilization of large-scale data  [3]. To date, several studies have proposed predictive models or developed algorithms aimed at identifying patients at risk of returning to the ED, including those at high risk  [4]. Moreover, EHRs often consist of different data type, for example, triage data or time series data. Given this, the researchers can utilize these types of data to explore various modeling approaches to predict the result we want.

Recent systematic reviews have further underscored the potential of deep learning in this field. Porto analyzed 60 primary studies and demonstrated that deep learning algorithms consistently outperform traditional methods in predicting emergency triage outcomes  [5]. Similarly, Kuo et al. conducted a meta-analysis on ED revisit prediction and emphasized that exploring deep learning models could provide deeper insights into performance variability and offer better trade-offs between sensitivity and specificity  [6]. Lee et al.  [7] conducted a scoping review summarizing existing machine learning models for predicting emergency department revisits. They found that most studies relied primarily on static features and traditional algorithms, with limited exploration of dynamic time-series data or deep learning approaches. These studies highlight both the growing maturity of deep learning applications and the remaining research gaps in integrating different types of clinical data for improved predictive accuracy.

Dynamic features, represented by time-series data such as vital sign trajectories, capture the temporal evolution of a patient’s condition. These temporal patterns can reveal physiological deterioration or incomplete recovery during the ED stay—subtle but critical information that static features alone cannot represent. Incorporating such dynamic data enables more accurate and timely prediction of adverse outcomes. Building upon these insights, the present study applies deep learning techniques to integrate static demographic data and dynamic clinical information into a robust hybrid model. This approach aims to enhance the prediction of high-risk ED revisits, addressing the limitations identified in prior reviews and contributing to the ongoing advancement of data-driven emergency care.

### Motivation

Unplanned return visits to the ED, particularly those classified as high-risk, are considered key quality indicators for assessing the performance of emergency care. They reflect not only the quality and appropriateness of initial care but also the overall burden on the ED system. One study proposed a statistical model to predict ED return visits within 72 hours  [4]. In addition to statical methods, several previous studies have proposed machine learning (ML) based models, like random forest, extreme gradient boosting (XGBoost)  [8], and autoregression models. As observed, the majority of previous research has relied on ML based methods to predict the task, while deep learning (DL) approaches have been explored less frequently.

The technology of deep learning has advanced considerably in the past decade, and numerous applications and there has been a rapid increase in studies and applications, especially in time series modeling in natural language processing (NLP). Given that clinical data, including those collected from ED, are typically composed of time series or tabular formats, researchers can leverage the characteristics of such data to achieve predictive goals by developing various models tailored to different data types. Temporal models have also been increasingly applied in the healthcare domain. For instance, RNN-based architectures, including Long Short-Term Memory (LSTM) networks, have been employed to predict unplanned readmissions to the intensive care unit (ICU)  [9]. Another study proposed an LSTM+CNN model to predict readmissions in urgent care settings using electronic health records  [10]. Beyond these deep learning approaches, feature engineering techniques have also shown promise in modeling temporal healthcare data. In particular, Porto and Fogliatto demonstrated that a feature engineering approach based on time-series signatures significantly improves the predictive performance of machine learning algorithms in forecasting patient arrivals to emergency departments  [11]. Importantly, their feature engineering strategy incorporated domain-specific clinical knowledge into the modeling process, closely aligning with the approach presented in this study. While deep learning has been explored for predicting ED readmissions, existing studies have primarily focused on long-term outcomes (e.g., 30-day readmission) and often fail to show substantial improvements over traditional machine learning (ML) approaches  [12]. The application of DL models to ED-related tasks remains rare, partly due to the typically short stay duration of ED patients. Consequently, most studies rely solely on static EHR data and tend to overlook time-series information, which is often short-term and fragmented in the ED setting.

The objective of this study is to construct a deep learning model for predicting short-term ED revisits, specifically targeting the early identification of high-risk patients shortly after discharge. By considering both time series and structured clinical data from EHR, we seek to improve the early identification of patients at risk of serious adverse outcomes upon return.

**Fig. 1:**
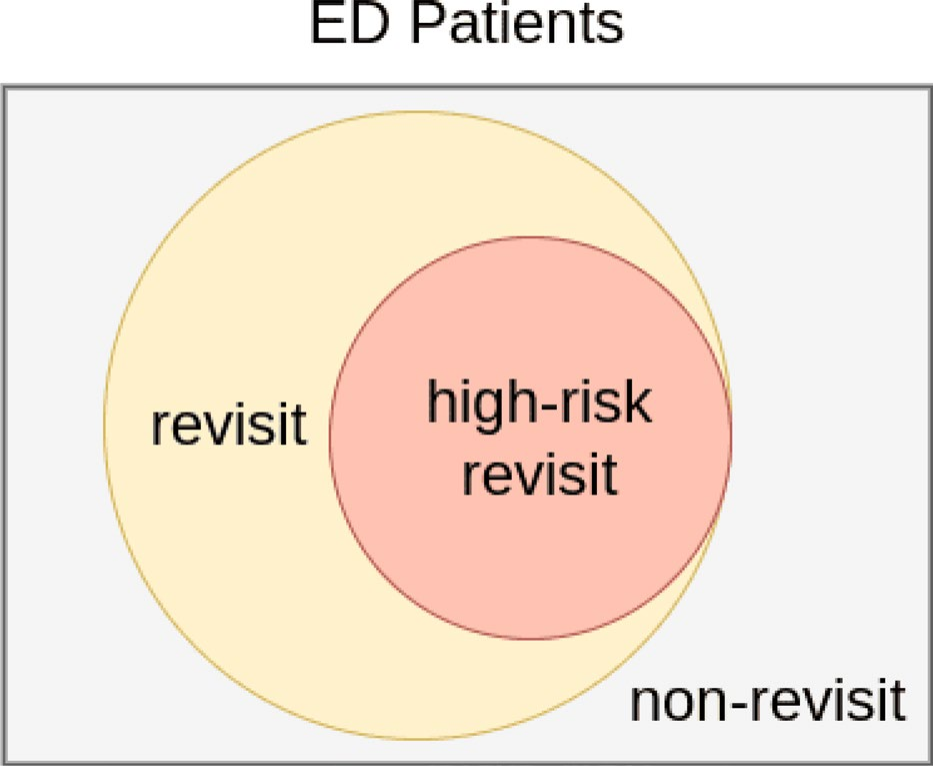
Illustration of the prediction tasks in this study

To construct and train a deep learning-based model, this study employs data obtained from the emergency department of National Taiwan University Hospital (NTUH), with datasets from different time periods used for internal validation. As illustrated in Fig. 1, the prediction task is divided into two subtasks: one aims to predict all revisit cases within three days of the index (first) ED visit, while the other focuses specifically on identifying high-risk revisits (revisits with intensive care unit admissions, cardiac arrest, or requiring emergency surgery).

### Challenges

#### Irregularity of measurements and missing values

In general, patient data in hospitals consist not only of static triage data collected at admission but also of dynamic measurements, such as vital signs, recorded over time. For inpatient settings, the duration of dynamic data collection is usually consistent. However, in emergency departments, patients have highly variable lengths of stay, resulting in irregularly sampled time-series data. Moreover, for patients with extremely short stays, the time intervals are insufficient for meaningful temporal patterns to emerge, often leading to severe data sparsity. These issues pose significant challenges for prediction tasks, as most temporal models require inputs of fixed length.

#### Limitations of static feature-based approaches

Machine learning-based methods remain the dominant approach for emergency department (ED) revisit prediction, largely due to their reliance on static features such as triage data. Prior studies have predominantly focused on these static variables, with limited incorporation of temporal features. However, patient’s return visits are typically influenced by multiple time-dependent factors. Neglecting the temporal dimension of clinical data may result in biased or incomplete representations of patient conditions, thereby limiting the effectiveness of predictive models.

#### Highly imbalanced data

In medical datasets, the number of positive samples associated with specific adverse events is typically rare, leading to significant class imbalance. In our NTUH dataset spanning from 2016 to 2019, the overall revisit rate is approximately 4.42%, while the high-risk revisit rate is only about 0.11%. This severe imbalance poses substantial challenges for seeking effective predictive model, particularly while aiming to achieve high model performance.

#### The value of short-term time series data

Medical data typically consist of both static and dynamic components. Static features include demographic information and triage assessments, while dynamic features refer to time-varying measurements such as vital signs. In particular, short time-series data, such as those collected during emergency department stays, offer critical temporal patterns that reflect rapid changes in a patient’s physiological condition. Despite their brevity, these temporal signals can provide valuable information for prediction tasks and should not be overlooked in the development of predictive models.

### Related works

Several recent studies have explored predictive models for hospital readmission using structured EHR or time series data with fixed sampling intervals. Table 1 shows the comparison between related works and our study. Sung et al.  [12] developed a prediction model for high-risk ED revisits within 72 hours, using a stacked ensemble of traditional machine learning models. Their input features included triage information and vital signs collected at the initial ED visit, represented as a single static record per patient.

**Table 1:** Comparison of our approach with existing methods

Paper	Using Feature Type	Dynamic Data Type	Hybrid Model	Length of Time Series	Deep Learning	Prediction Target
Sung et al. [12]	Static	–	–	–	–	ED Revisit
Kim et al. [13]	Static + Dynamic	Lab results Medication	–	3 time steps	$\checkmark$	Hospital Readmission
Lopez et al. [14]	Static	–	–	–	$\checkmark$	Hospital Readmission
Ashfaq et al. [15]	Static + Dynamic	Diagnoses Lab results Medication	–	≥48 hr	$\checkmark$	Hospital Readmission
Tang et al. [16]	Static + Dynamic	Diagnoses X-ray image	$\checkmark$	≥48 hr	$\checkmark$	Hospital Readmission
**Ours**	Static + Dynamic	Vital signs	$\checkmark$	≤24 hr	$\checkmark$	ED Revisit

Kim et al.  [13] proposed a deep learning model based on Gated Recurrent Units (GRU) to predict hospital readmission among patients with heart failure. Their model incorporated both static and temporal features from the EHR, capturing sequential dependencies in the patient records. Similarly, Lopez et al.  [14] trained both ML and DL models using structured EHR data to identify patients with asthma or COPD who were likely to experience recurrent exacerbations and rehospitalizations.

Ashfaq et al.  [15] proposed a deep learning framework for predicting unplanned 30-day readmissions by combining expert-defined features with sequential patterns extracted from EHR. Their method emphasized preserving the temporal order of patient events. A multimodal spatiotemporal graph neural network (MM-STGNN), designed for predicting 30-day all-cause hospital readmissions, was proposed by Tang et al.  [16]. Their approach modeled inter-admission relationships using graph structures that captured similarity across patient visits based on clinical features.

While these studies demonstrate the potential of deep learning in modeling readmissions, most of them rely on regularly sampled time series data or structured tabular EHR records. In contrast, our study focuses on the ED setting, where time series data, particularly vital signs, are irregularly sampled and often limited to short observation windows. Unlike previous works that tend to discard such short or irregular sequences, we include all available patient data, regardless of length. Furthermore, we explicitly integrate both static triage information and dynamic temporal features, and extract additional statistical indicators such as the delta and standard deviation of vital signs to enhance model performance and interpretability.

### Objectives

As mentioned in previous section, our research faces several challenges. This section details the techniques used to overcome major challenges inherent in our dataset, namely missing data, hybrid feature representations, class imbalance, and non-uniform time series sampling.

#### Concerning data length irregularity

In the data preprocessing stage, we align irregular time series data to a fixed length. For example, when a patient’s ED stay lasts only for 3 hours, we apply padding to align the sequence with the standardized 24-hour input length. To address missing values, we use both forward and backward imputation to fill the sequences. By retaining the temporal dynamics of feature changes, this approach allows the model to effectively learn clinically relevant patterns, such as sudden spikes or drops in vital signs.

#### Concerning limitations of static data

In addition to analyzing patients’ static triage information, we also incorporated dynamic data into model training. In contrast to machine learning approaches which utilize only static features, our method integrates dynamic features enabling the model to capture dynamic aspects of patient conditions that are often overlooked, thereby improving predictive performance.

#### Concerning data imbalanced

To address the issue of extreme class imbalance, we apply data augmentation techniques to static and dynamic data, respectively. For static data, we employ oversampling to increase the number of positive samples to a level comparable with the negative samples. For dynamic data, we use window shifting and conventional time series augmentation methods  [17], such as noise injection and scaling. These techniques are applied during the training phase to augment the number of positive samples, with the effect of improving the model’s ability so as to detect rare events.

#### Concerning short-term time series

To enhance the model’s capacity to capture clinically meaningful patterns associated with ED revisit risk, we aim to design a hybrid representation scheme that effectively integrates both static features and short-term temporal signals. Static features provide a snapshot of the patient’s baseline condition at the time of admission, while short-term temporal features derived from vital sign fluctuations offer dynamic insights into the patient’s physiological trajectory. By jointly modeling these data types, the proposed representation aims to capture a more comprehensive and nuanced understanding of patient status, with the effect of improving the accuracy and robustness of ED revisit prediction.

## Methods

### System overview

**Fig. 2:**
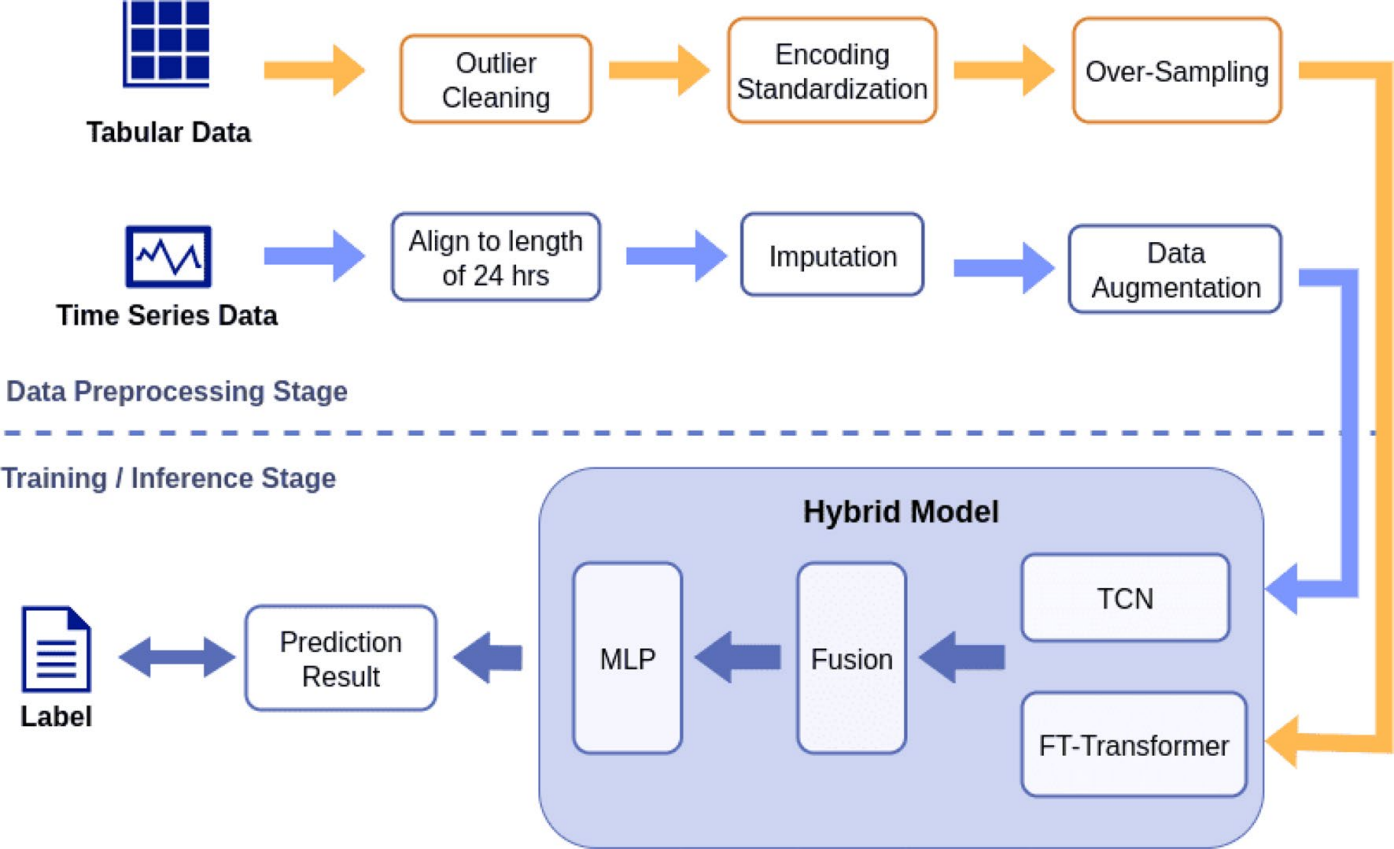
System overview

In this study, the proposed prediction system is designed in two stages: a data preprocessing stage and a training/inference stage. Figure 2 illustrates the system overview of our research.

The upper part of the figure illustrates the data preprocessing stage. The raw input is divided into two categories: tabular triage data and time series data, where the latter represents the vital sign fluctuations of patients during the ED stay. These two types of data are subsequently used as the inputs for the system’s static and dynamic features, respectively. We first perform outlier removal to clean abnormal values in the dataset. For time series data with irregular lengths, we align all sequences to a fixed length of 24 hours prior to discharge, then fill in missing values by imputation. The static features undergo label encoding and normalization. To address the class imbalance, we apply data augmentation and oversampling techniques. Finally, the processed static and dynamic features are used as separate inputs to the corresponding modules in the hybrid architecture.

The lower portion of the figure depicts the model training and prediction process for identifying whether a patient will return to the emergency department, and if so, whether that return constitutes a high-risk visit. Our system is composed of two modules, whose outputs are integrated through a weighted fusion mechanism to produce the final prediction. During the training phase, the predicted outcomes are evaluated against the ground-truth labels, and the resulting loss is minimized via gradient descent to iteratively update the model parameters.

In the inference stage, the model output is transformed into a probability score ranging from 0 to 1, indicating the likelihood of a revisit. A score closer to 1 implies a higher probability of return, while a score closer to 0 indicates a lower risk. This predicted probability can serve as a reference to support clinical decision-making. The details of additional implementation are described in the subsequent section.

### Data description

In contemporary medical research, electronic health records (EHR) are widely utilized as a valuable source of clinical data. In this study, we used the NTUH ED dataset from 2016 to 2019 for model training and testing, whereas the dataset from 2020 to 2022 is reserved for internal validation in the study.

Upon arrival at the emergency department, clinical staff first record the patient’s demographic information and triage details. Vital signs are then measured and documented, with subsequent recordings taken at irregular intervals depending on the patient’s condition. Since these measurements are manually entered, the timing of observations is not regular, resulting in time series that differ in both length and sampling frequency. Our dataset includes two types of features: static features that remain constant throughout the observation period, and dynamic features that reflect changes over time.

#### Static features

Static features refer to patient information documented at the time of arrival in the emergency department. These include background information and triage data, which are documented only once and therefore represented in tabular format. The NTUH dataset includes a total of 19 static features, comprising 5 numerical and 14 categorical variables, as detailed in Table 2. Each patient record contains a single instance of static information, collected at the triage station upon arrival. These features include demographic and clinical information such as age, triage level, and mode of arrival.

**Table 2:** List of static features and their types

Static Feature	Description
Age	Age of the patient
Weekend triage	Whether the triage occurred on a weekend
Department	Subdivision of ED
Ambulance	Whether the patient arrived at the ED by ambulance
Sex	Gender of the patient
Season	Season during which the patient visited the ED
Dayzone	Time slot of the patient’s arrival (day, evening, night)
Dayzone discharge	Patient discharge time slot
Weekend discharge	Whether the discharge occurred on a weekend
Triage level	Triage category according to the Taiwan Triage and Acuity Scale
ED length of stay	Duration of patient stay in the ED
Household income	Patient’s estimated household income based on postal code
Major disease	Whether the patient has a major chronic disease
Judgement code new	Triage code reflecting initial clinical assessment of the patient
Marital status	Patient’s marital status
Employment status	Patient’s employment status
Nationality	Nationality of the patient
Sex of treating physician	Treating physician’s gender
Age of treating physician	Treating physician’s age

#### Dynamic features

Dynamic features refer to continuously monitored vital sign values recorded during a patient’s stay in the ED. These data are structured as time series, capturing temporal fluctuations in the patient’s physiological status. In our dataset, six types of vital signs are collected as dynamic features, as listed in Table 3. Due to the typically short and irregular duration of patient stays in the emergency department, most previous studies on revisit prediction have primarily focused on analyzing triage data alone. In contrast, our study incorporates both triage (static) data and time series (dynamic) data to provide a more comprehensive assessment.

**Table 3:** List of dynamic features with types and units

Dynamic Feature	Unit
Heart rate	beats/min
Diastolic blood pressure	mmHg
Oxygen saturation	%
Body temperature	^∘^C
Respiratory rate	breaths/min
Systolic blood pressure	mmHg

### Data preprocessing

This section describes the data preprocessing procedures employed to convert raw inputs into a standardized format appropriate for model development and evaluation. Since all patient measurements are manually recorded, occasional inaccuracies or implausible values are inevitable. To reduce the impact of extreme errors, we first applied outlier removal. Specifically, in the time series vital signs data, values that fall outside the physiological range are considered abnormal and set to null. The normal ranges of vital signs are listed in Table 4. These missing values are subsequently addressed during the imputation phase.

**Table 4:** Normal range thresholds for dynamic features

Dynamic Feature	Normal Range
Heart rate	[0, 300)
Respiratory rate	[0, 50)
Oxygen saturation	[0, 100]
Diastolic blood pressure	[0, 300)
Body temperature	[0, 50)
Systolic blood pressure	[0, 300)

#### Dynamic feature input

To prepare time series data for model input, we begin by addressing the issues of non-uniform sequence lengths and sporadic missing values, in order to prepare the data for input into the TCN. Our preprocessing procedure consists of three main steps: Hourly Division, Fixed Series Length, and Data Imputation. Each step is described in the following sequel.

##### Hourly division

**Fig. 3:**
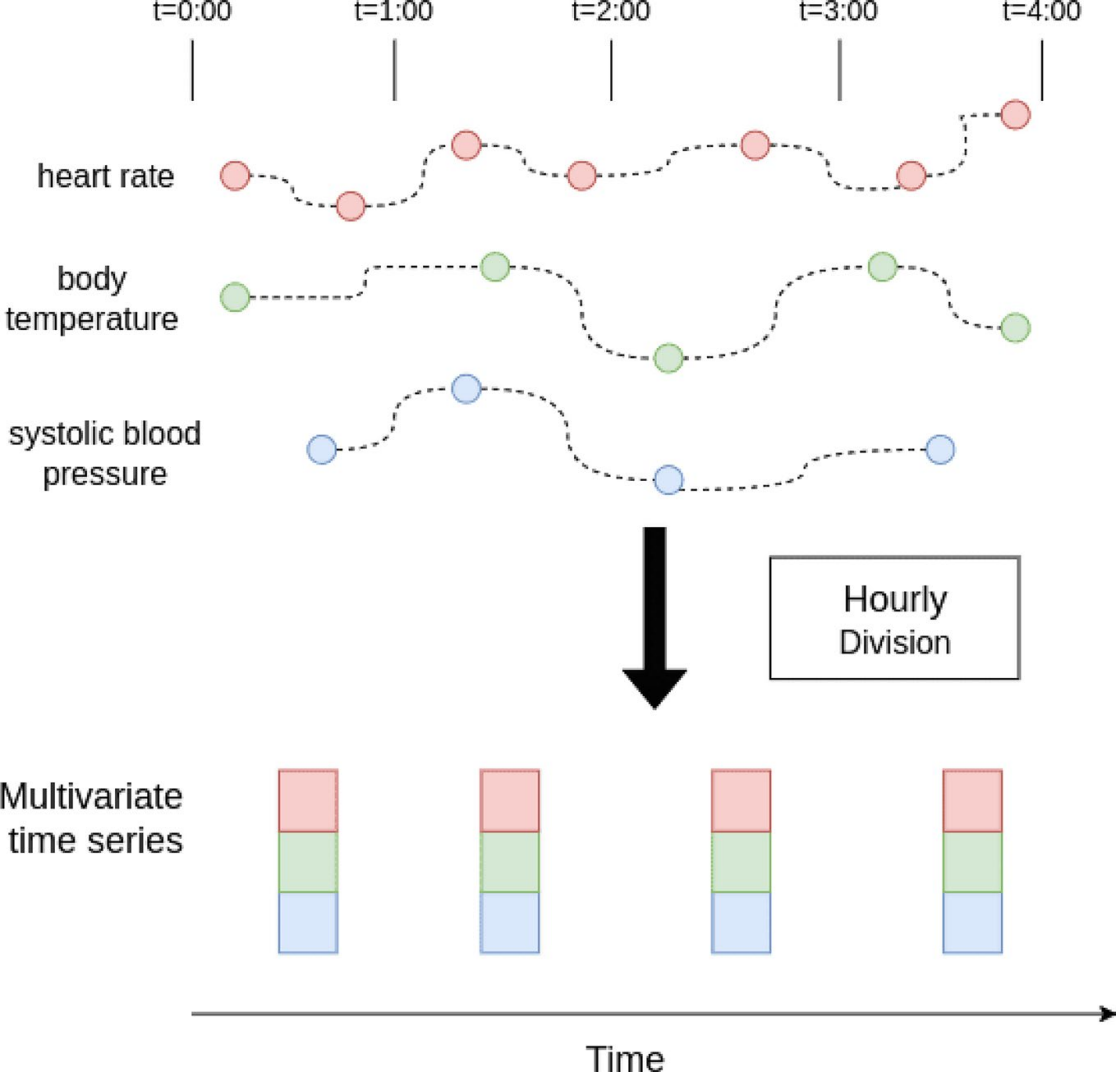
Example of hourly division

To enable the model to capture temporal correlations between measurements, it is essential to align the irregularly sampled timestamps. This problem can be addressed using the hourly division strategy, as proposed in prior work  [18]. In this approach, the time series is segmented into fixed one-hour intervals. If multiple measurements of the same vital sign are taken within a given hour, we computed their average to represent that vital sign of the patient during that hour. As a result, the number of hours becomes the sequence length, allowing the model to analyze temporal patterns all with consistent intervals. As illustrated in Fig. 3, the example produces a multivariate time series with a length of four hours and three dynamic features.

##### Fixed series length

**Fig. 4:**
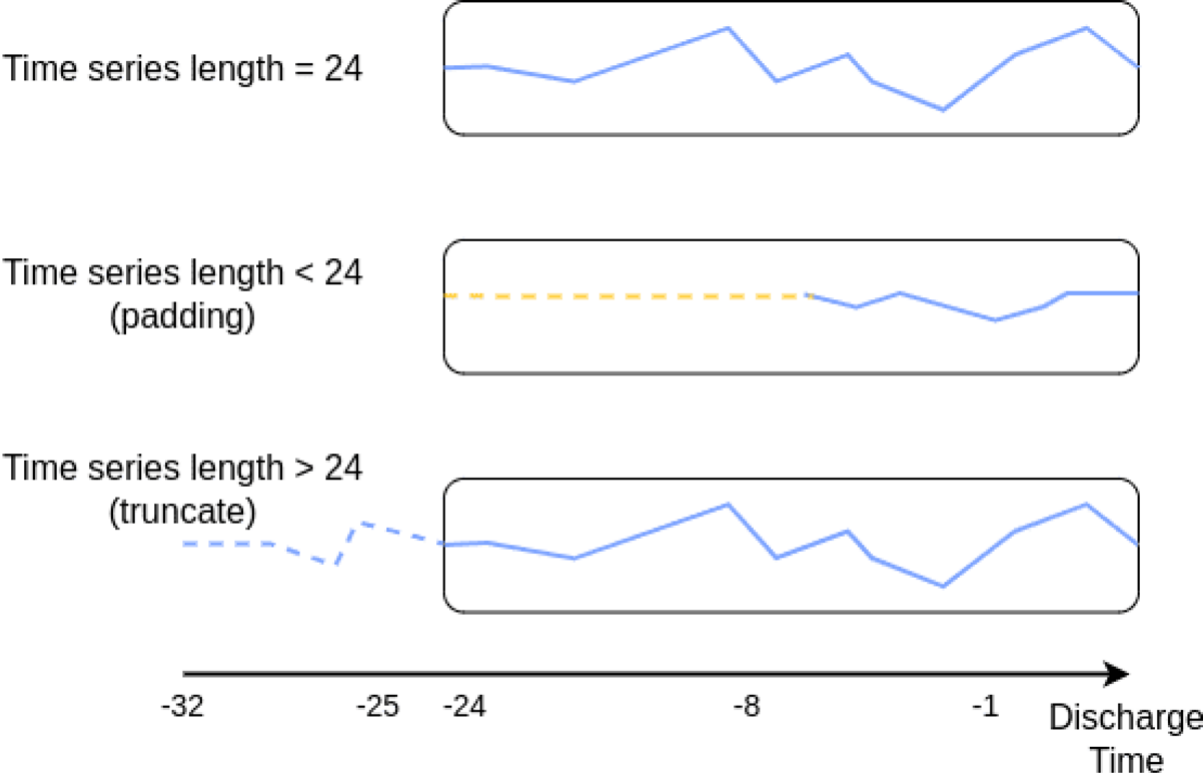
Example of time series alignment

To enable the model to effectively learn from dynamic data, each time series must be adjusted to a fixed length. In our study, we selected the 24-hour period preceding discharge, resulting in sequences of length 24 time steps (i.e., up to the final hour at discharge, where one time step is taken as one hour in the following text). This window is intended to capture the patient’s physiological status leading up to their departure from the emergency department. For patients with data period shorter than 24 hours of data, we applied zero-padding to extend the period to the desired length. Conversely, for patients with data period longer than 24 hours of records, the period is truncated to retain only the most recent 24 hours. Figure 4 illustrates how sequences of varying lengths are processed using this approach.

##### Data imputation

In the emergency department, vital signs are typically measured at intervals of three to four hours. As a result, dividing the data into hourly segments often leads to a high proportion of missing values within the time series. In addition, null values also arise from the earlier removal of outliers. While many recent studies have explored imputation methods for time series data, which includes both statistical and deep learning approaches  [19], most deep learning methods rely on long input sequences to effectively learn temporal patterns. In our setting, the sequences are relatively short, and after alignment to a fixed length, the missing rate becomes significantly high. Under such conditions, using complex imputation models may not only fail to improve data quality but also distort the original time trends.

In this study, we adopted a simple and widely used strategy by applying forward-filling followed by backward-filling. This two-step approach is designed to preserve temporal consistency and reduce the risk of information leakage. Forward-filling uses the last observed value to fill subsequent missing entries, which aligns with the natural temporal direction of the data. Backward-filling is applied only when the beginning of a sequence contains missing values that cannot be imputed by forward-filling. This approach helps ensure completeness while maintaining the integrity of the temporal structure and reducing potential bias from missing data.

**Figure:**
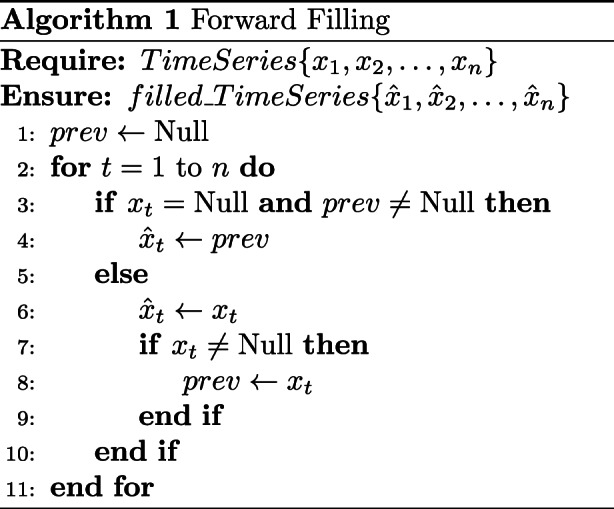

**Figure:**
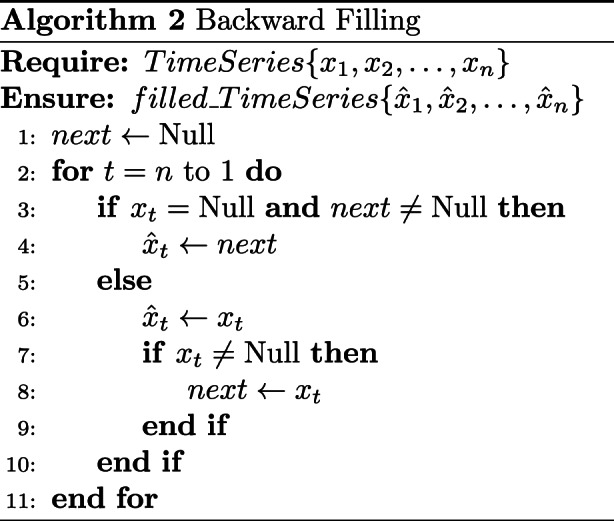

**Fig. 5:**
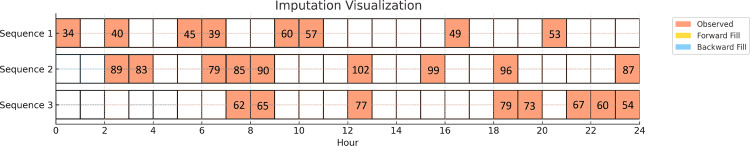
Example of time series before imputation

**Fig. 6:**
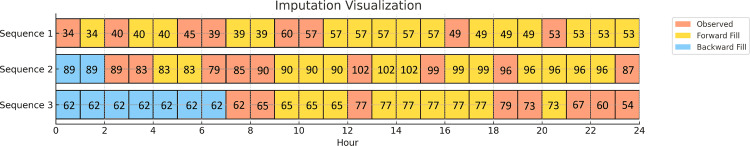
Example of time series after imputation

Figures 5 and 6 illustrate the time series data before and after imputation, respectively, highlighting the effect of the applied imputation strategy.

### Tabular data

**Fig. 7:**
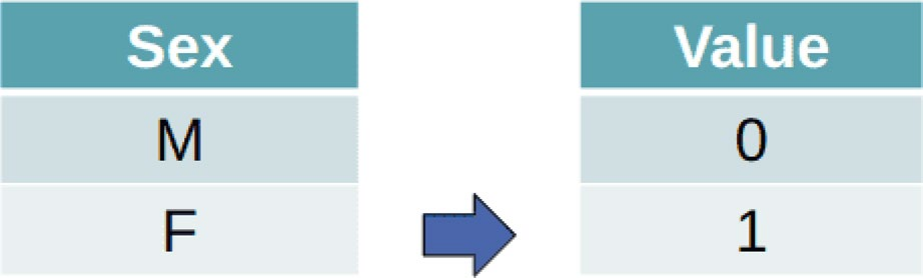
Label encoding of “sex” feature

All static features are represented in tabular form. To prepare these features for input into the FT-Transformer, we applied a series of preprocessing steps. Some of the categorical features are originally non-numeric, so we converted them into integer representations ranging from 0 to *n* − 1 using label encoding. Figure 7 shows an example of label encoding applied to the feature, say, *sex*.

**Fig. 8:**
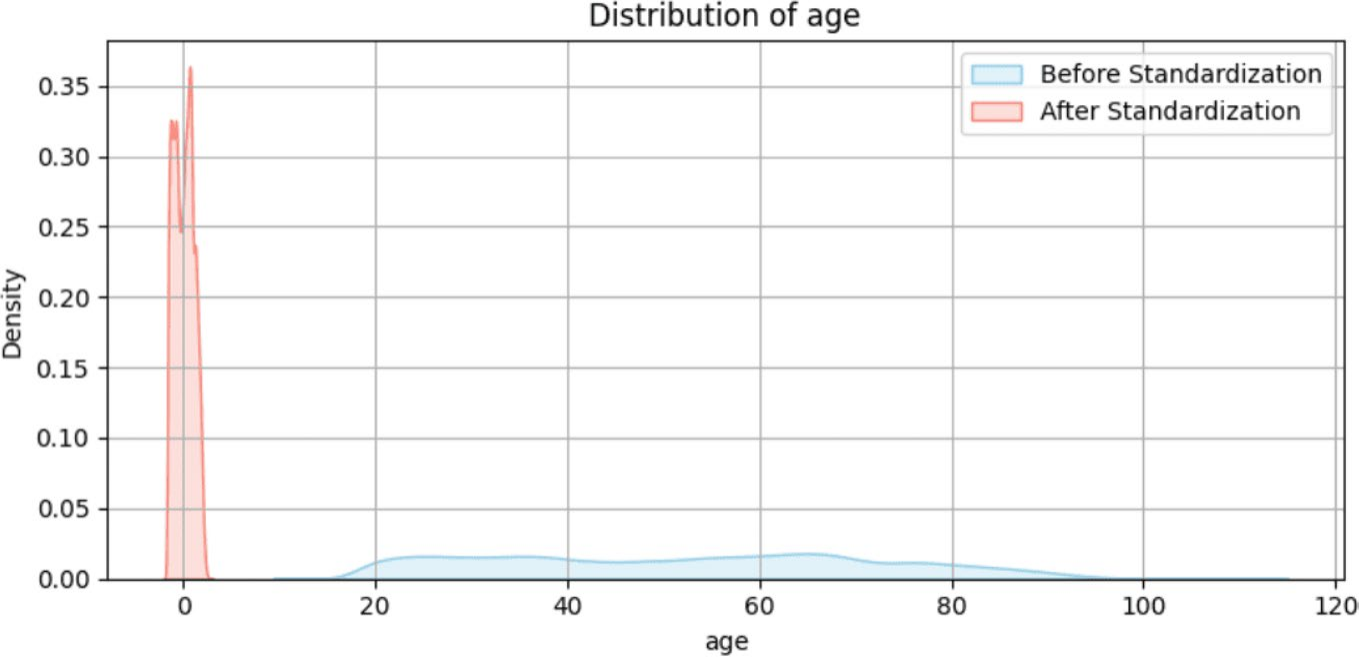
Standardization of “age” feature

For continuous numerical features, we applied standardization to facilitate effective processing by the FT-Transformer. Since the FT-Transformer employs linear projections to map static numerical features into a common latent space, it is important that the input features are on comparable scales. Without normalization, features with larger numeric ranges may disproportionately influence the model’s learned representations. Specifically, we used z-score normalization on all numerical features, transforming each value to have a zero mean and unit variance. This is achieved by subtracting the mean and dividing by the standard deviation of each numerical feature, calculated from the data. Such normalization not only accelerates convergence during training but also improves model stability and performance. Figure 8 illustrates the normalization process for the feature *age*.

### Data augmentation

Given the highly imbalanced nature of our dataset, where positive samples are extremely scarce, it is challenging to directly apply deep learning techniques of data augmentation. To address this issue, we adopted the traditional strategies to increase the number of positive samples for model training. This section presents the data augmentation strategies implemented to mitigate the issue of severe class imbalance. Our approach consists of three primary components: window shifting, random transformation, and over-sampling.

#### Window shifting

The first augmentation method is window shifting. Since the original time series has been preprocessed into a fixed-length sequence of 24 time steps, applying a sliding window over the data becomes straightforward. To address the class imbalance issue, we applied this augmentation only to positive samples. Specifically, for patients who returned to the emergency department, we applied window shifting to increase the number of positive training samples.

**Fig. 9:**
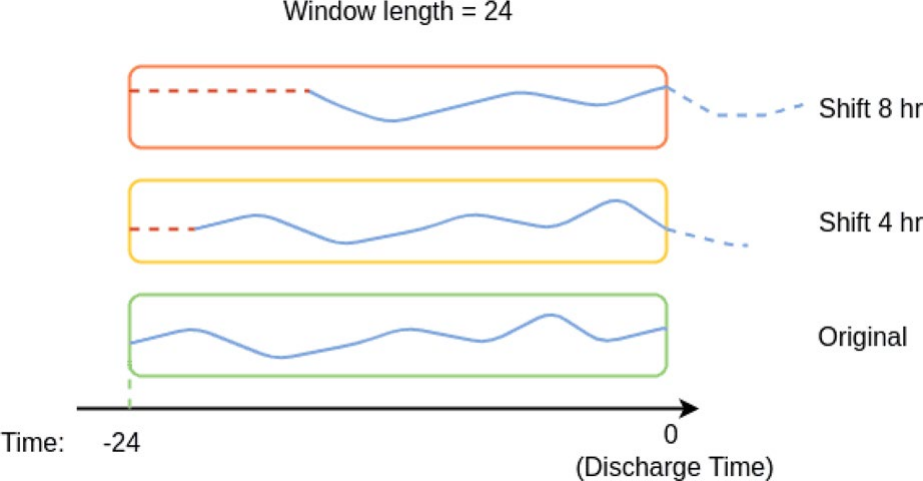
Example of window shifting

In our implementation, the window size is fixed at 24 time steps, and the sliding step is 1 hour. Each shift moves the window one hour earlier relative to the discharge time, resulting in a new time series data with 24 hours length, i.e., the 1st hour data are dropped, but the last hour data are imputed. As illustrated in Fig. 9, different colors represent different time windows. For example, the original window includes the 24 hours prior to discharge, where *t* = 0 denotes the time of discharge and *t* = −24 hr is the end time of the time window. A window shifted one hour earlier covers the interval from *t* = −25 hr to *t* = −1 hr before discharge, and so on. In total, we applied 8 shifts, covering time intervals from *t* = −32 hr to *t* = −8 hr prior to discharge. Just like the case with one hour shift, the cases with other different hour shifts will also generate new time series data still with 24 hr length by corresponding data dropping as well as forward-filling imputation.

This strategy enables the model to learn from multiple temporal segments of the same patient, effectively expanding the set of positive samples and improving balance in the training data. The impact of different shift counts is further evaluated in the ablation study section.

#### Random transformation

After applying window shifting, we further augment the resulting positive samples to increase the quantity and diversity of training data. In order to achieve this, we adopted a set of commonly used time series data augmentation techniques, collectively referred to as random transformations  [17]. The following paragraphs describe the specific methods employed in this study.

**Fig. 10:**
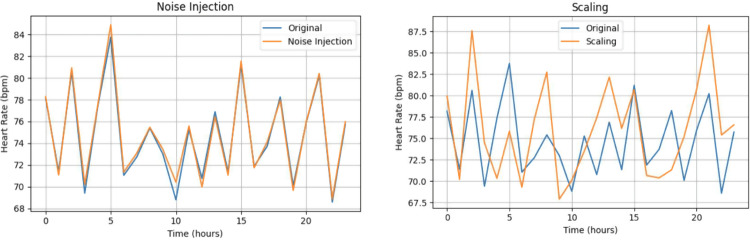
Example of data augmentation

Refer to Fig. 10, the first method is noise injection, which adds small random Gaussian noise to the original values. This simulates measurement error or natural physiological variability, allowing the model to learn from subtle fluctuations in the sequence. In our implementation, the noise standard deviation was set to 12% of the feature’s original standard deviation. This range was chosen to introduce minor variability without distorting the underlying physiological trend. We confirmed through preliminary experiments that larger magnitudes of noise degraded model performance, while smaller magnitudes showed negligible benefit.

Next is scaling, in which the entire sequence is multiplied by a randomly sampled factor in the range of 0.9 to 1.1. This simulates patients whose overall vital sign levels are systematically higher or lower than average. The scaling range was determined based on observed inter-individual variability in vital signs, ensuring that the augmented samples remain physiologically plausible. We also compared model performance with and without scaling augmentation, and found that this transformation provided a modest improvement in validation AUROC while maintaining stable training behavior.

#### Over-sampling

For static data, the number of positive samples is also limited. However, compared to time series data, augmenting tabular data is relatively straightforward. To address class imbalance, we employed an over-sampling technique that increases the proportion of positive samples. The augmented static features are then provided as input to the static branch of the model.

### Hybrid model architecture

**Fig. 11:**
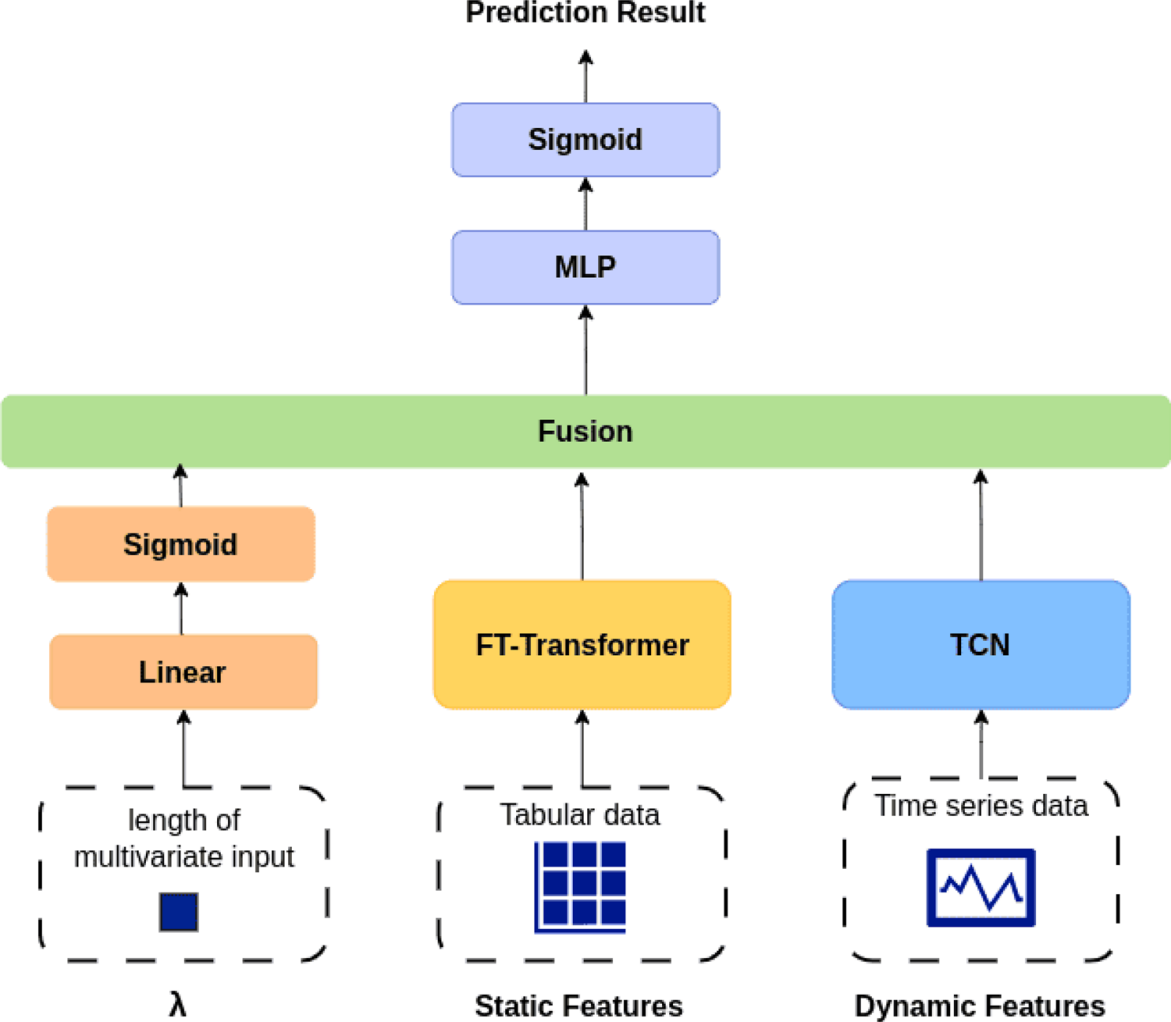
Model architecture

This section introduces the architecture of the proposed hybrid model, as illustrated in Fig. 11. The following subsections provide detailed descriptions of each component, including the Temporal Convolutional Network (TCN), the FT-Transformer, the fusion mechanism, and the loss function.

#### Temporal convolutional network

In time series modeling tasks, previous studies have demonstrated that the Temporal Convolutional Network (TCN)  [20] achieves strong performance across various domains. Unlike recurrent neural networks (RNNs), which rely on sequential memory structures, TCN adopts a convolution-based architecture that leverages causal convolutions and dilated convolutions to process sequential inputs. In contract to RNNs, which compute outputs step-by-step and are constrained by sequential dependencies, TCN enables parallel computation across time steps, significantly improving training speed, which is an important advantage when working with large-scale patient datasets. Moreover, the use of dilated convolutions allows the model to learn long-range dependencies with fewer layers while maintaining the same input and output sequence lengths. Finally, compared to RNN-based models, which are prone to issues such as gradient vanishing or exploding, TCN incorporates residual blocks, offering better training stability and faster convergence.

#### FT-Transformer

The input to the FT-Transformer is the static data, which includes triage information recorded upon patient arrival at the emergency department. FT-Transformer is a Transformer-based tabular model and is well-suited for handling mixed-type features, including both categorical and numerical variables. By embedding each feature as a token, the model effectively captures complex feature interactions, making it particularly appropriate for modeling diverse static variables in clinical datasets. Additionally, the feature-level attention mechanism contributes significantly to the model’s interpretability, which is beneficial in medical applications.

In the preprocessing stage, we applied label encoding to categorical features and normalization to numerical features. This allows the feature tokenizer to generate embeddings in a consistent format, enabling the model to learn meaningful representations of individual features. Categorical features such as judgement-code, marital-status-code, triage, diagnosis-serious, and ambulance are embedded through a lookup table, whereas numerical features such as age and house income are projected through a linear embedding layer. The resulting feature tokens are then processed by the attention layers of the Transformer encoder, which learns the relationships among the features.

In the experimental section, we compared FT-Transformer with TabNet to assert its performance on modeling of static features.

#### Fusion

**Fig. 12:**
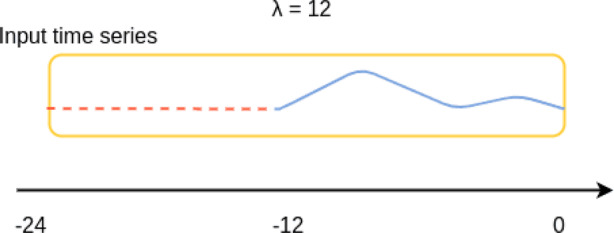
Example of *λ*

In the fusion stage, we concatenated the outputs from both the dynamic and static branches. While the Temporal Convolutional Network (TCN) effectively captures short- and long-term temporal dependencies in dynamic time-series data, it is inherently limited to sequential patterns and lacks the ability to model complex relationships among static variables. Conversely, the FT-Transformer excels at learning nonlinear feature interactions and global dependencies among static attributes but cannot directly capture temporal dynamics. By fusing the representations from these two complementary architectures, the model leverages both the temporal sensitivity of the TCN and the cross-feature interaction capability of the FT-Transformer, thereby achieving a more comprehensive understanding of patient states. This hybrid design also enhances generalization, as each branch compensates for the other’s representational bias. Since padding operations during sequence modeling may distort the original data distribution, we incorporated a length indicator to help the model focus on the valid information. Inspired by  [21], we introduce *λ*, which represents the actual length of the original input sequence before padding, as shown in Fig. 12.

During the fusion stage, *λ* is used as a weighting factor applied to the model output, guiding the model to recognize the effective portion of the sequence. This design mitigates the impact of padding-induced noise and improves the model’s robustness during training.

**Fig. 13:**
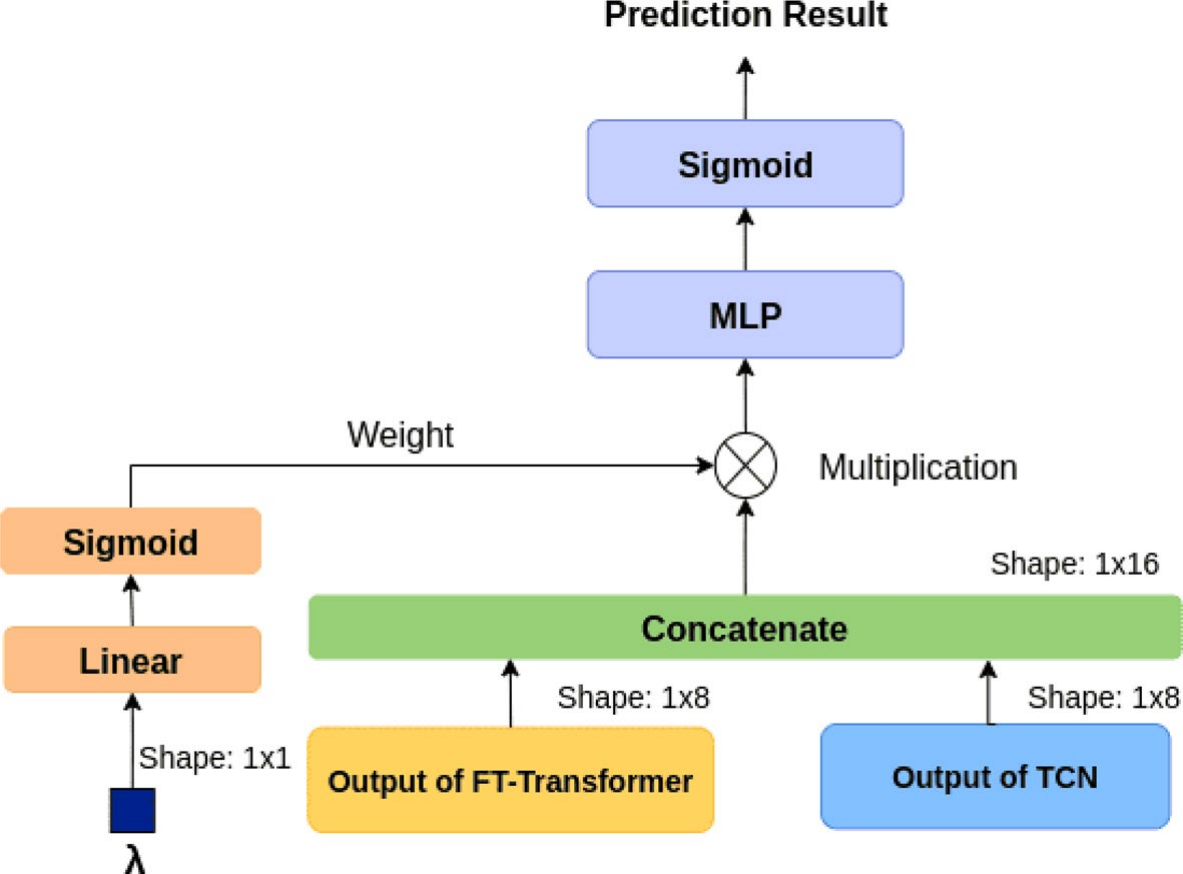
Fusion

The scalar is first passed through a linear layer followed by a sigmoid activation. The resulting weight is then used to scale the concatenated output from the two model branches, producing the final prediction, which is shown in Fig. 13. This mechanism enables adaptive fusion between static and dynamic representations, allowing the model to adjust its reliance on temporal or static information according to the completeness and duration of each patient’s record. Consequently, the model better adapts to patients with varying lengths of ED stay and achieves improved predictive performance across diverse clinical scenarios.

#### Loss function

To address the severe class imbalance in our dataset, we adopted Focal Loss as the objective function to improve the model’s focus on hard-to-classify samples. This approach reduces the overwhelming influence of the majority class by dynamically down-weighting the well-classified examples that focusing the training on hard, misclassified instances.

Focal Loss is defined as follows:

$$\text{FL}(p_t) = -\alpha (1 - p_t)^\gamma \log(p_t)$$

where $p_t$ is the predicted probability of the true class, $\alpha$ is the weighting factor, and $\gamma$ is the focusing parameter.

In our implementation, we set $\alpha = 0.4$ and $\gamma = 2.0$. The loss is computed based on the final output of the fusion module, which integrates both static and dynamic representations. This formulation enhances the model’s ability to detect rare but clinically significant outcomes.

#### Prediction

Finally, we describe our prediction procedure. After processing the input through both the TCN and FT-Transformer modules, the outputs are fused and combined using the aforementioned weights *λ*. The fused representation is then passed through a multi-layer perceptron (MLP)-based classifier to generate the final prediction. This prediction is a continuous value between 0 and 1, where values closer to 1 indicate a higher likelihood that the patient will return to the emergency department.

## Results and discussion

### Experimental setup

The experimental environment consists of an Intel i7-10700K processor, 32 GB RAM, and an NVIDIA RTX 3090 GPU. Model training is carried out using PyTorch 1.13.1 with Python 3.8.18. For training, we used a batch size of 32 and the Adam optimizer with learning rate of $5 \times 10^{-4}$. In addition, we implemented an early stopping mechanism, which terminates training if the AUPRC does not improve for more than five consecutive epochs. Table 5 lists the detailed system setting.

**Table 5:** Experimental environment Description

Component	Specification
OS	Ubuntu 20.04
GPU	NVIDIA RTX 3090
CPU	Intel Core i7-10700K
RAM	32 GB

### Dataset

In our experiments, we used a dataset collected from the emergency department (ED) of National Taiwan University Hospital (NTUH), which includes patients who revisited the ED after discharge. The NTUH is a tertiary academic medical center with approximately 2400 beds and 100,000 ED visits per year.

The dataset consists of two time periods: 2016–2019 and 2020–2022. For the 2016–2019 dataset, there are a total of 252,171 patient records, including 11,150 revisit cases, among which 275 are classified as high-risk. This dataset is used for model development, with 70% allocated for training, 15% for validation, and 15% for testing. As for the 2020–2022 dataset, it contains 136,732 patient records in total, including 7707 revisit cases, of which 281 are high-risk. This dataset is used as an internal validation set to further assess the model’s generalizability.

**Fig. 14:**
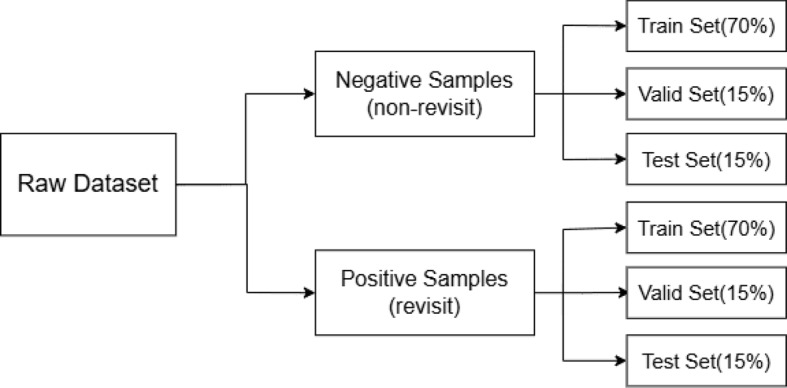
Data distribution of all revisit population

**Fig. 15:**
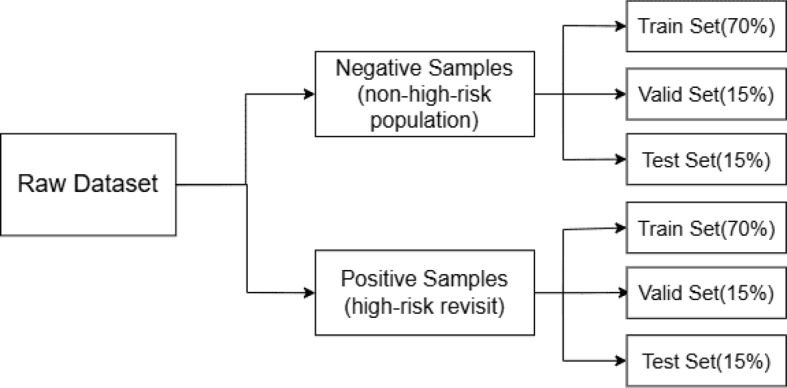
Data distribution of high-risk revisit population

To ensure accurate proportion of positive cases across all subsets in different cases, we stratified the data by revisit status before splitting. The detailed data partitioning strategies are illustrated in Figs. 14 and 15, which show the data distribution for general revisit prediction and high-risk revisit prediction, respectively.

As shown in Table 6, the proportion of positive samples in the NTUH datasets remains extremely low across different time periods. In the 2016–2019 dataset, the proportion of general revisit cases is roughly at 4.42%, whereas the proportion of high-risk revisit cases is only 0.1%, indicating a severe class imbalance. In the 2020–2022 dataset, the rate of general revisit cases increases to 5%, whereas that of high-risk revisit cases increases to 0.2%. This increase is likely associated with the COVID-19 pandemic, during which emergency department revisit rates experienced a marked rise  [22]. Additionally, we calculated the median sequence length for each patient’s stay. The results indicate that most patients had very short stays in the ED.

**Table 6:** Comparison of dataset characteristics

Characteristic	NTUH 2016–2019	NTUH 2020–2022
Positive Sample Proportion (revisit)	4.42%	5%
Positive Sample Proportion (high-risk revisit)	0.11%	0.2%
Sequence Length of Vital Signs (Median)	2 (hours)	1 (hour)

### Validation approach

In this study, we adopted a temporal data-splitting approach for model validation rather than a conventional k-fold cross-validation. According to recent methodological recommendations, particularly in the context of clinical prediction modeling, temporal validation is considered a more appropriate strategy when longitudinal data are available. This approach evaluates the models performance on a future time frame that is distinct from the training period, thereby providing a more realistic assessment of generalizability. As highlighted by Hond et al.  [23] and Moons et al.  [24], temporal or time-split validation serves as an intermediate and more robust method between internal and external validation. Moons et al. further noted that randomly splitting a single dataset constitutes an inefficient form of internal validation, whereas temporal validation better reflects real-world prediction scenarios by assessing performance across different time periods. Therefore, we followed this recommended practice rather than applying k-fold cross-validation in the present study.

### High-risk revisit prediction

In this section, we first evaluated the model performance on the high-risk revisit prediction task. We conducted experiments separately for the multivariate time series model and the tabular model. In addition to testing on the held-out test set from the NTUH 2016–2019 ED dataset, we further assess model generalizability by performing internal validation on the entire NTUH ED dataset collected between 2020 and 2022.

#### Results on NTUH 2016–2019 dataset

In this subsection, we evaluated the performance of our developed predictive model, focusing on the module of handling the multivariate time series data in our proposed hybrid model on the NTUH test set. Specifically, we compared our employed TCN with GRU and LSTM respectively serving as two other competitive baselines. Table 7 illustrates the results that TCN consistently outperforms the others across all evaluation metrics, achieving AUROC of 0.8453 and AUPRC of 0.0935.

**Table 7:** Predictive performance of module of handling time series data on high-risk ed revisits (NTUH 2016–2019 test set)

Model	AUROC	AUPRC	Precision	Recall	F1-score
High-risk revisit prediction (base rate of precision: 0.01)					
GRU+FT-Transformer	0.7990	0.0479	0.0329	**0.5952**	0.0623
LSTM+FT-Transformer	0.8452	0.0712	0.0354	**0.5952**	0.0668
**TCN+FT-Transformer**	**0.8453**	**0.0935**	**0.0428**	**0.5952**	**0.0799**

Likewise, we evaluated our developed predictive model but focusing on the module of handling the static feature data in our hybrid model also on the NTUH 2016–2019 dataset. Here, we specifically compared our employed FT-Transformer with two other competitive baselines: one is called Logistic Regression, which only considers static feature data and the first recorded vital sign after admission, whereas the other is TabNet with the earlier TCN. As shown in Table 8, the FT-Transformer demonstrates superior performance across all metrics and achieves nearly five times improvement in precision over the base rate. In contrast, logistic regression, which relies solely on static features, performs significantly worse than hybrid models that incorporate both static and dynamic inputs.

**Table 8:** Predictive performance of module of handling static feature data on high-risk ed revisits (NTUH 2016–2019 test set)

Model	AUROC	AUPRC	Precision	Recall	F1-score
High-risk revisit prediction (base rate of precision: 0.01)					
Logistic Regression (static only)	0.4971	0.0097	0.0075	**0.2143**	0.0145
TabNet+TCN	0.7815	0.0385	0.0387	**0.5952**	0.0727
**FT-Transformer+TCN**	**0.8453**	**0.0935**	**0.0428**	**0.5952**	**0.0799**

#### Internal validation on NTUH 2020–2022 dataset

We further conducted internal validation for the high-risk revisit prediction task using the NTUH 2020–2022 dataset, where all patient records are included for evaluation. We first evaluated our employed module TCN which handles the multivariate time series data similar to the experiment conducted in Subsection 4.3.1, with the results summarized in Table 9. Although GRU slightly outperforms TCN in terms of precision and F1-score, TCN still achieves the best performance in terms of AUROC and AUPRC.

**Table 9:** Internal validation of TCN handling the multivariate time series data on high-risk revisit prediction using the NTUH 2020–2022 dataset

Model	AUROC	AUPRC	Precision	Recall	F1-score
High-risk revisit prediction (base rate of precision: 0.002)					
GRU+FT-Transformer	0.7691	0.0097	**0.0057**	**0.6014**	**0.0113**
LSTM+FT-Transformer	0.7053	0.0050	0.0047	**0.6014**	0.0093
**TCN+FT-Transformer**	**0.7976**	**0.0103**	0.0056	**0.6014**	0.0112

Likewise, Table 10 presents the evaluation results of FT-Transformer which handles static feature data similar to what has been done on Subsection 4.3.1 but now on the NTHU 2020–2022 dataset. While the FT-Transformer shows slightly lower precision and F1-score than TabNet, it consistently achieves higher AUROC and AUPRC values, demonstrating its robustness across key metrics.

**Table 10:** Internal validation of FT-Transformer on high-risk revisit prediction using the NTUH 2020–2022 dataset

Model	AUROC	AUPRC	Precision	Recall	F1-score
High-risk revisit prediction (base rate of precision: 0.002)					
Logistic Regression (static only)	0.7648	0.0060	0.0057	**0.7046**	0.0093
TabNet+TCN	0.7673	0.0087	**0.0059**	**0.6014**	**0.0117**
**FT-Transformer+TCN**	**0.7976**	**0.0103**	0.0056	**0.6014**	0.0112

To sum up, the above results indicate that our proposed hybrid model which integrates TCN and FT-Transformer provides stable and strong performance for the high-risk revisit prediction task across different datasets collected over different time periods.

### General revisit prediction

In this section, we evaluated model performance on the general revisit prediction task. As in previous experiments shown in Section 4.3, we assessed both the module, namely, TCN which handles the multivariate time series data and another module, namely, FT-Transformer which handles static feature using the NTUH 2016–2019 test set and the NTUH 2020–2022 internal validation set.

#### Results on NTUH 2016–2019 dataset

Recall that 15% of the entire NTUH 2016–2019 dataset is taken as the test set. Table 11 shows the evaluation results of our predictive model with TCN in our proposed hybrid model handling the multivariate time series data for the general revisit task. Like before, we compared our model with two other competitive baselines repectively with GRU and LSTM. While GRU achieves slightly higher AUPRC than TCN, the TCN outperforms both GRU and LSTM in other metrics, namely, AUROC, precision, and F1-score.

**Table 11:** Predictive performance of module of handling time series data on general ed revisits (NTUH 2016–2019 test set)

Model	AUROC	AUPRC	Precision	Recall	F1-score
General revisit prediction (base rate of precision: 0.042)					
GRU+FT-Transformer	0.7220	**0.2094**	0.0918	**0.5998**	0.1592
LSTM+FT-Transformer	0.7214	0.1900	0.0903	**0.5998**	0.1569
**TCN+FT-Transformer**	**0.7250**	0.2005	**0.0984**	**0.6002**	**0.1690**

We further evaluated our developed predictive model but focusing on the module of handling the static feature data in our hybrid model on the NTUH 2016–2019 dataset. Logistic Regression is also used as a baseline that relies solely on static features. As shown in Table 12, the FT-Transformer consistently outperforms the baselines across all metrics, highlighting its effectiveness in modeling static clinical data for general revisit prediction.

**Table 12:** Predictive performance of module of handling static feature data on general ed revisits (NTUH 2016–2019 test set)

Model	AUROC	AUPRC	Precision	Recall	F1-score
General revisit prediction (base rate of precision: 0.042)					
Logistic Regression (static only)	0.4995	0.0441	0.0431	**0.2887**	0.0750
TabNet+TCN	0.7161	0.1843	0.0875	**0.5998**	0.1527
**FT-Transformer+TCN**	**0.7250**	**0.2005**	**0.0984**	**0.6002**	**0.1690**

#### Internal validation on NTUH 2020–2022 dataset

We then evaluated the general revisit prediction task on the NTUH 2020–2022 internal validation set. Table 13 summarizes the performance of our employed module TCN which handles multivariate time series data similar to the experiment conducted in Subsection 4.4.1. GRU achieves slightly higher AUROC and AUPRC than TCN in this task. However, when considering all metrics comprehensively, TCN remains the most stable and consistent model across evaluations.

**Table 13:** Internal validation of TCN handling the multivariate time series data on general revisit prediction using the NTUH 2020–2022 dataset

Model	AUROC	AUPRC	Precision	Recall	F1-score
General revisit prediction (base rate of precision: 0.05)					
GRU+FT-Transformer	**0.7321**	**0.1657**	0.1257	**0.6000**	0.2078
LSTM+FT-Transformer	0.7282	0.1496	0.1240	**0.6000**	0.2056
**TCN+FT-Transformer**	0.7300	0.1537	**0.1260**	**0.6000**	**0.2082**

Likewise, Table 14 presents the evaluation results of tabular models on the NTUH 2020–2022 dataset, following the same experimental setting described in Subsection 4.4.1. The FT-Transformer consistently achieves strong performance across all metrics, reaffirming its effectiveness in modeling static clinical data for general revisit prediction.

**Table 14:** Internal validation of FT-Transformer on general revisit prediction using the NTUH 2020–2022 dataset

Model	AUROC	AUPRC	Precision	Recall	F1-score
General revisit prediction (base rate of precision: 0.05)					
Logistic Regression (static only)	0.6585	0.1016	0.1012	0.5253	0.1697
TabNet+TCN	0.7212	0.1447	0.1228	**0.6000**	0.2038
**FT-Transformer+TCN**	**0.7300**	**0.1537**	**0.1260**	**0.6000**	**0.2082**

Overall, the results confirm that TCN and FT-Transformer consistently deliver strong and stable performance across both test and validation datasets for the general revisit task.

Although the model’s precision appears numerically low, this should be interpreted in the context of the outcome’s base rate. In terms of clinical applicability, the practical meaning of this precision can be better understood by translating it into the expected number of alerts. For instance, in a real-world setting such as the National Taiwan University Hospital, approximately 300 emergency visits occur per day. Given a precision of 0.0428 for high-risk patients, the model would generate alerts for only about 13 individuals per day. This alert volume is relatively small and would not be expected to cause alarm fatigue among healthcare personnel. Instead, it could feasibly support clinicians by flagging a manageable number of potentially high-risk patients for further evaluation.

To further assess the robustness and stability of the proposed model, we conducted an additional experiment simulating post-pandemic changes in revisit rates due to an aging population. Specifically, we increased the proportion of high-risk revisits to 0.003 and general revisits to 0.06. Under these modified class distributions, the model achieved an AUPRC of 0.0075 and a precision of 0.005 for high-risk revisits, and an AUPRC of 0.1349 and a precision of 0.1238 for general revisits. These results indicated that the model maintained relatively stable predictive performance with varying outcome prevalence.

### Ablation studies

#### Impact of architectural modules on prediction performance

This section investigates the impact of individual model components on overall predictive performance. We conducted ablation studies by systematically removing the TCN module, the FT-Transformer module, and the scalar fusion weight *λ* employed in the integration stage. Experiments are conducted on the NTUH 2016–2019 test set, targeting the high-risk revisit prediction task.

As shown in Table 15, removing any of the components results in a noticeable drop in performance. Excluding the FT-Transformer leads to the most significant decrease in AUPRC, nearly 58%, highlighting its critical role in modeling static features. Removing the TCN also degrades overall performance, confirming the importance of dynamic feature modeling. Interestingly, excluding the scalar input *λ* yields a slight increase in AUROC, but still results in a 32% reduction in AUPRC. This suggests that incorporating LoS(length-of-stay)-dependent weighting through *λ* is beneficial for improving the model’s precision in identifying rare revisit cases.

**Table 15:** Ablation study on architectural design on NTUH 2016–2019 test set. *λ* denotes the length of multivariate input (without padding)

Model Variant	AUROC	AUPRC	Precision	Recall	F1-score
**ALL**	0.8453	**0.0935**	**0.0428**	**0.5952**	**0.0799**
- FT-Transformer	0.7441	0.0388	0.0262	**0.5952**	0.0502
- TCN	0.8151	0.0740	0.0323	**0.5952**	0.0612
- *λ*	**0.8470**	0.0628	0.0411	**0.5952**	0.0769

#### Impact of maximum backward window shift on model performance

We further investigated the effect of different maximum window shifting lengths on model performance. Specifically, we experimented with maximum shifting lengths of 4, 6, 8, 12, 16, and 25 hours to observe how the size of the observation window affects the results. Here, each length above indicates the maximum number of one-hour shifts applied to the input sequence; for example, a 4-hour shifting means the window is shifted up to four times, each by one hour.

**Fig. 16:**
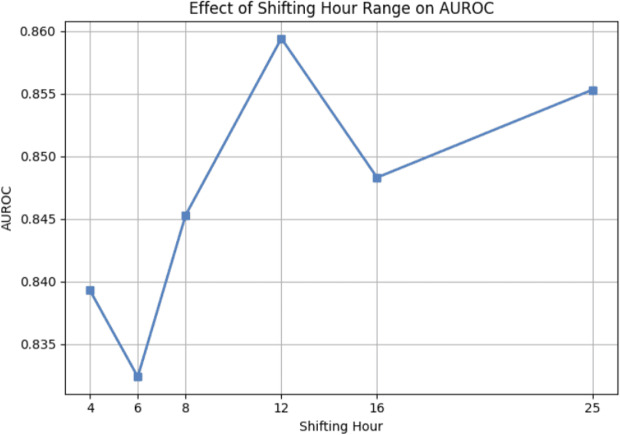
Performance on window shifting maximum size for AUROC

**Fig. 17:**
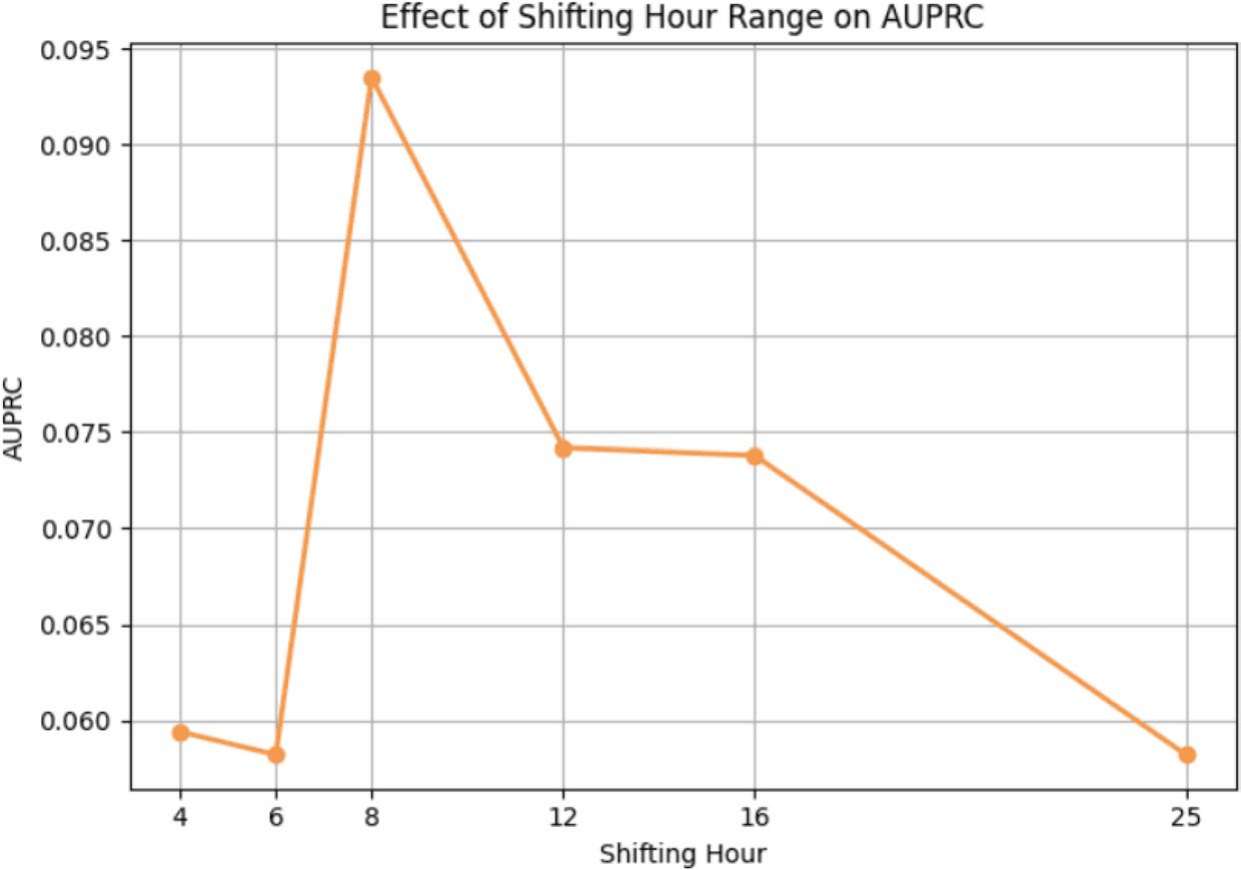
Performance on window shifting maximum size for AUPRC

Figures 16 and 17 illustrate the changes in AUROC and AUPRC, respectively, across different shifting lengths. The model achieves the highest AUROC when the window is shifted by 12 hours, whereas the best AUPRC is observed when shift to 8 hours. Considering both results, although the AUROC increase from 8-hour setting to 12-hour setting by approximately 0.015, the AUPRC drops by about 0.02, which apparently suggests that the 8-hour setting is more favorable. Therefore, we selected the 8-hour window as the primary configuration for window shifting in our study.

### Interpretability

#### Feature importance

To better understand how the model predicts patient revisit outcomes, we conducted feature importance interpretation and case analysis for both dynamic and static features. We conducted the experiments on the high-risk ED revisit dataset from NTUH 2016–2019 test set. For dynamic features, we adopted a gradient-based saliency method  [25]. Specifically, for each individual sample, we performed backpropagation from the model output to the input time series features and compute the absolute value of gradient at each time step. The mean absolute gradient across all time steps is used as the saliency score for each feature. We then averaged the saliency scores across all samples to obtain a global estimate of dynamic feature importance. For static features, we directly extracted the attention weights from the FT-Transformer. The average attention score of each feature is used to assess its relative importance.

**Fig. 18:**
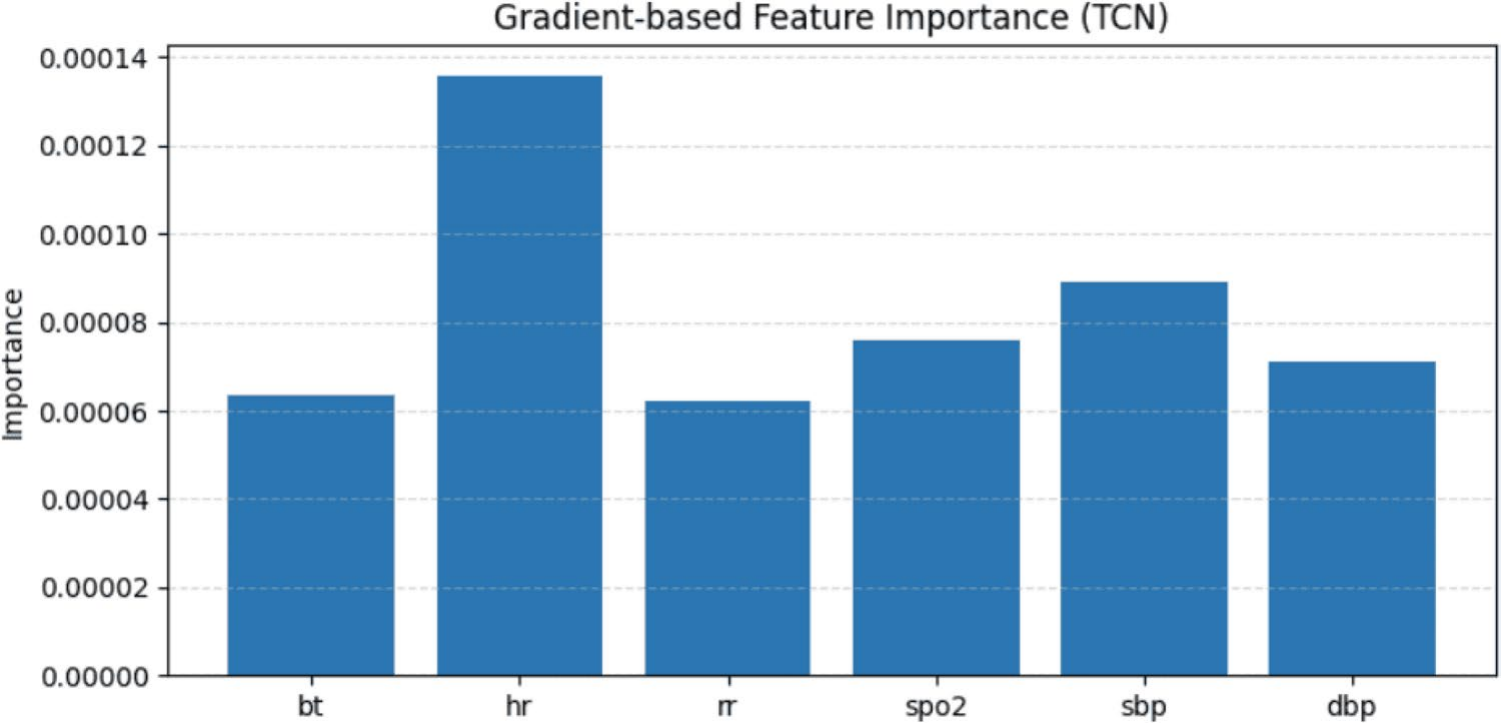
Dynamic features importance

We conducted feature importance analysis for both types of input features. To estimate the importance of dynamic features, we adopted a gradient-based saliency method like before. For each individual sample, we performed backpropagation from the model output to the input time series features, and compute the average absolute value of the gradient across all time steps as the saliency score for each feature. By averaging the saliency scores across all samples, we obtained a global estimate of feature importance, reflecting how sensitive the model’s predictions are to changes in each time series feature. In Fig. 18, heart rate exhibits the highest contribution, followed by systolic blood pressure (SBP).

**Fig. 19:**
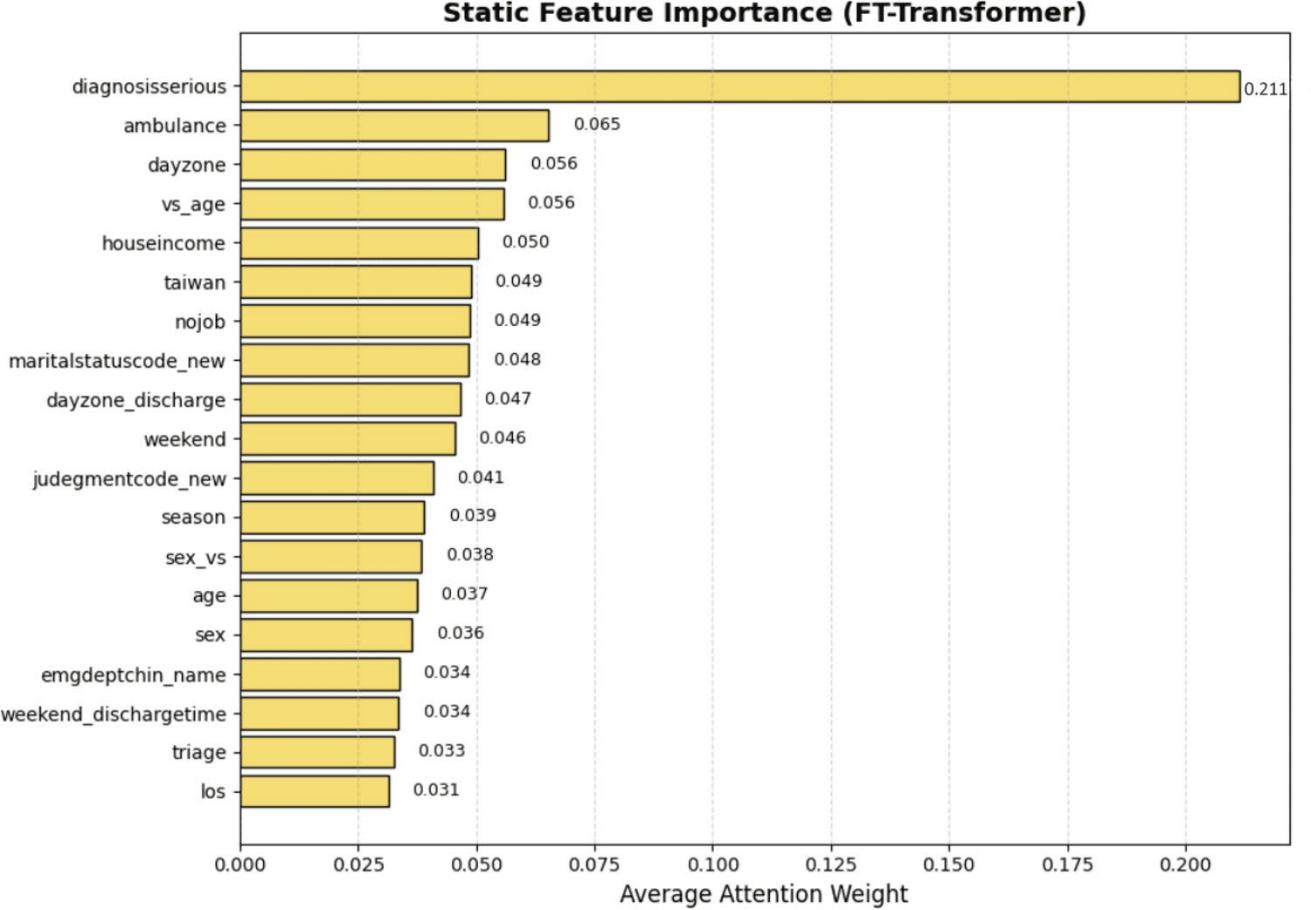
Static features importance

For static features, we utilized the self-attention weights from the FT-Transformer encoder to assess feature importance. Specifically, we extracted the attention weights assigned by the final layer’s [CLS] token to each input feature token. These weights reflect the degree of focus the model places on each static feature when aggregating information for prediction. As such, they serve as indicators of the model’s perceived relevance of each static feature to the prediction task. The yellow bars in Fig. 19 depict the importance of static features. Major diseases emerges as the most influential feature, followed by ambulance usage and dayzone.

These findings suggest that for future applications in clinical settings, more attention should be given to the effectiveness of Transformer-based models for tabular data. In addition, incorporating short-term time series vital signs could provide further support for clinical decision-making.

#### Case study

In this subsection, we conducted a case study on a patient labeled as a high-risk revisit from the NTUH 2016–2019 test set. As in previous analyses, we examined the contribution weights of both dynamic and static features to the model’s prediction. For this patient, the model produced a predicted probability of 0.6293, indicating a greater than 50% likelihood of being a high-risk revisitor (base rate = 0.01).

**Fig. 20:**
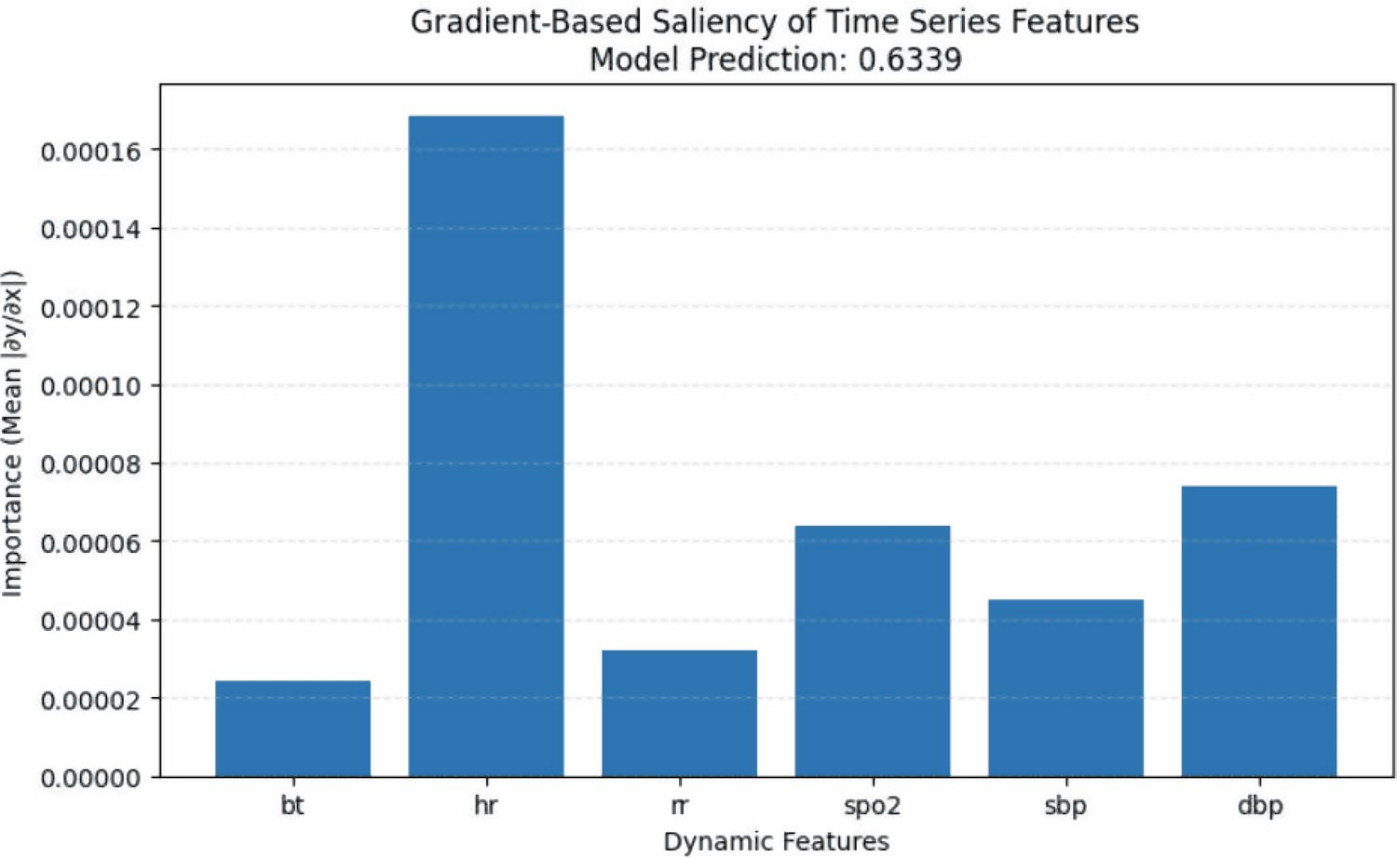
Dynamic Features Importance of a High-Risk Patient

**Fig. 21:**
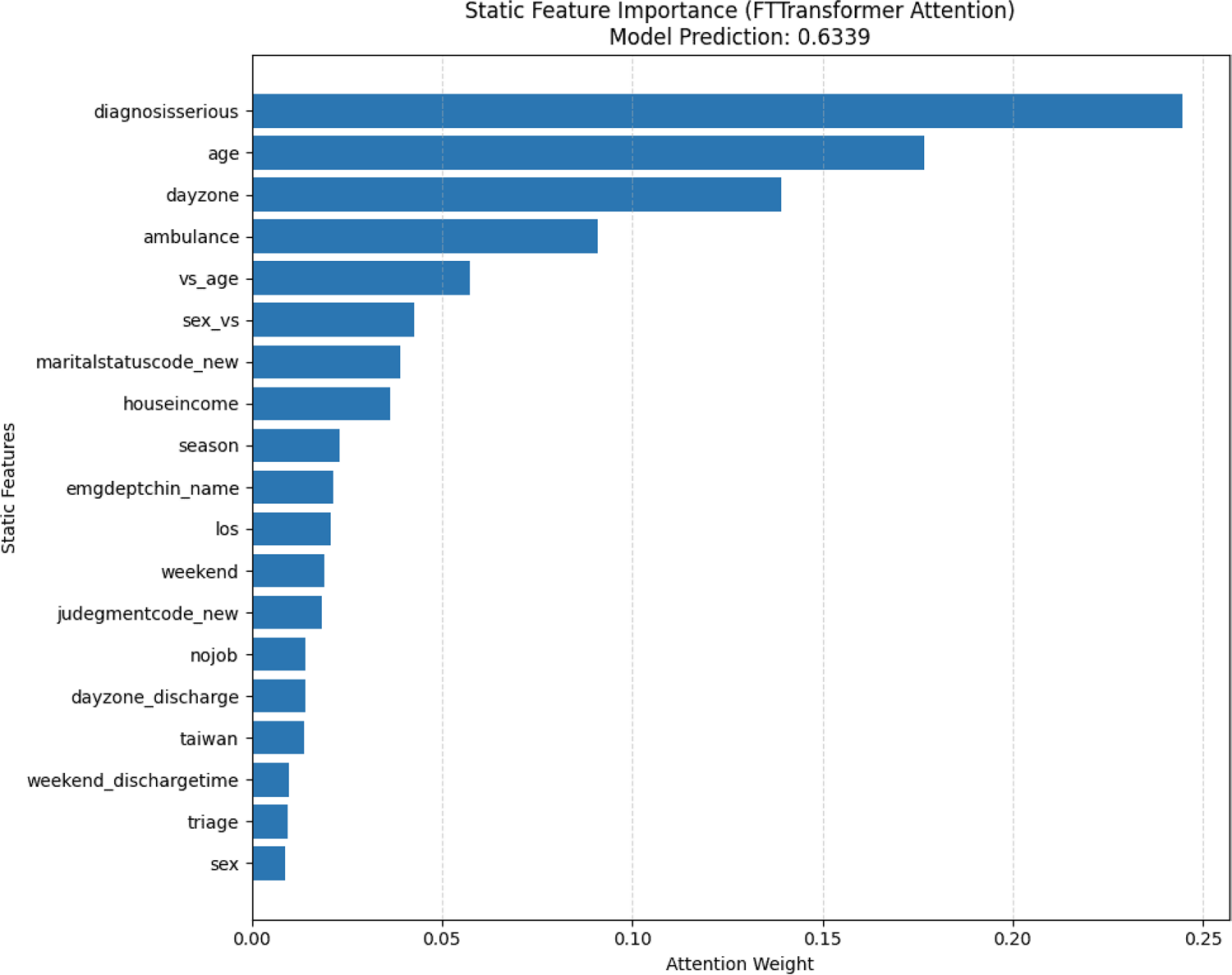
Static Features Importance of a High-Risk Patient

As shown in Fig. 20, fluctuations in heart rate have the greatest influence on the model’s decision, followed by diastolic blood pressure (DBP) and $\text{SpO}_2$. In Fig. 21, the most influential feature is the Diagnosis serious indicator (whether the diagnosis was classified as severe), followed by age, dayzone, and ambulance usage.

**Fig. 22:**
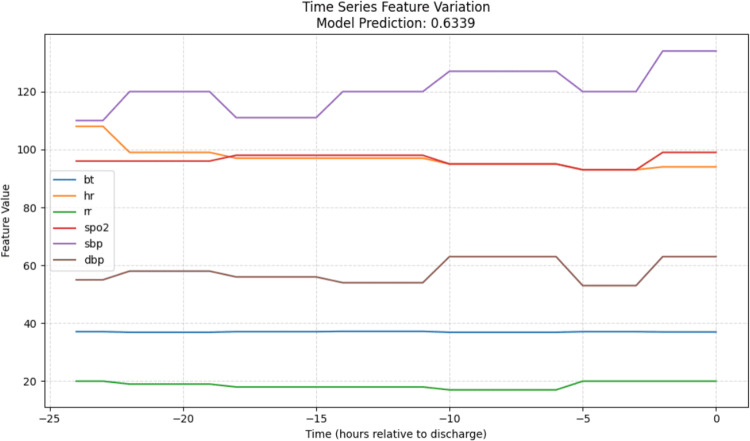
Time series feature variation of a high-risk Patient’s vital signs

Figure 22 illustrates the temporal patterns of vital signs for this patient. Notably, the patient exhibits consistently elevated heart rate (HR) readings, ranging from 90 to 100 bpm, throughout the entire stay. This observation aligns with the earlier feature importance analysis, suggesting that patients with persistently high heart rates are more likely to experience high-risk revisits.

## Conclusion

Emergency department (ED) revisit is widely regarded as an important indicator of emergency care quality. However, researches in this area remain limited in terms of applying deep learning techniques, especially with respect to irregularly time series data. Moreover, few studies have jointly considered both triage information and vital signs with temporal fluctuations.

In this study, we developed a deep learning–based prediction model for ED revisit, employing a hybrid architecture that integrates static and dynamic features. Through the designed preprocessing strategies, our model addresses the challenges of irregular time series and missing values. Model performance was evaluated using an internal validation dataset.

Our model achieved an AUROC of 0.8453 and an AUPRC of 0.0935 (base rate = 0.01) in the high-risk revisit prediction task, as well as an AUROC of 0.7250 and an AUPRC of 0.2005 (base rate = 0.042) in the general revisit prediction task. Both results significantly outperformed the static-only logistic regression baseline. We also utilized the emergency department dataset from NTUH spanning 2020 to 2022 as our internal validation cohort. Our model achieved an AUROC of 0.7976 and an AUPRC of 0.0103 (base rate = 0.002) for the high-risk revisit prediction task, and an AUROC of 0.7300 and an AUPRC of 0.1537 (base rate = 0.05) for the general revisit prediction task. Both results significantly outperformed the static-only logistic regression baseline. The an AUPRC has increased from 0.0288 to 0.0935, and the precision has improved from 0.0281 to 0.0428, representing an increase to roughly 1.52 times.

Although removing the FT-Transformer leads to the greatest performance drop in the ablation study, feature importance analysis shows that the TCN contributes more. We hypothesize that the FT-Transformer provides generalizable and stable information (e.g., age, triage level), serving as a foundational base, whereas the TCN plays a more decisive role in crucial cases through temporal patterns that reflect patient deterioration or recovery trajectories. Additionally, we performed feature importance analysis demonstrating that heart rate exerts the strongest influence on prediction outcomes. We also conducted case studies to identify key patient characteristics that contributed to the model’s predictions.

Despite the promising results, this study has certain limitations. The model was primarily developed and validated using data from the National Taiwan University Hospital (NTUH), which may limit its generalizability to other countries or regions. Differences in healthcare systems, clinical practices, disease prevalence, and patient demographics across regions could influence model performance. Future research should therefore aim to validate and adapt the proposed framework using multi-center or international datasets to assess its robustness and applicability in diverse clinical environments.

Overall, our findings demonstrate that the proposed model effectively integrates dynamic time-series and static tabular features, yielding reliable and generalizable performance in predicting emergency department revisits. This highlights the potential of fusing different types of clinical data sources to improve early risk identification. The results also suggest that time-aware, hybrid deep learning architectures can serve as a valuable tool in clinical decision-making, particularly in settings where both temporal patterns and static characteristics are critical for patient outcomes.

Future work will focus on deploying the model in a real-world emergency department setting to evaluate its performance in clinical practice. Further improvements can also be made in the processing of temporal data and model design, to enhance the model’s ability to learn from and utilize sequential information more effectively. Moreover, leveraging insights from feature importance analysis could help adjust prediction weights and improve interpretability in clinical settings. Finally, we could further assess the robustness of our model with domain shifts; for example, by testing the model on data from other hospitals or evaluating performance over time as clinical practices evolve.

## Data Availability

The datasets used and/or analyzed during the current study are available from the corresponding author on reasonable request.

## Abbreviations

ED: Emergency Department
EHR: Electronic health records
IHCA: In-Hospital Cardiac Arrest
DL: Deep Learning
FT-Transformer: Feature Tokenizer Transformer
TCN: Temporal Convolutional Network
NTUH: National Taiwan University Hospital
SBP: Systolic blood pressure
DBP: Diastolic blood pressure
HR: Heart rate
AUROC: The area under the receiver operating characteristic curve
AUPRC: The area under precision recall curve

## References

[1] Ministry of health and welfare, Taiwan: 2022 health and welfare report – emergency department statistics. Accessed: 2025-04-08. 2022. https://dep.mohw.gov.tw/dos/lp-5103-113-xCat-y111.html.

[2] Sung C-W, Lu T-C, Fang C-C, Lin J-Y, Yeh H-F, Huang C-H, Tsai C-L. Factors associated with a high-risk return visit to the emergency department: a case-crossover study. Eur J Emerg Med. 2021;28(5):394–401. 10.1097/mej.0000000000000851. 10.1097/MEJ.0000000000000851 34191766

[3] Si Y, Du J, Li Z, Jiang X, Miller T, Wang F, Jim Zheng W, Roberts K. Deep representation learning of patient data from electronic health records (ehr): a systematic review. J Biomed Inf. 2021;115:103671. 10.1016/j.jbi.2020.103671. 10.1016/j.jbi.2020.103671 PMC11290708 33387683

[4] Pellerin G, Gao K, Kaminsky L. Predicting 72-hour emergency department revisits. Am J Emerg Med. 2018;36(3):420–24. 10.1016/j.ajem.2017.08.049. 10.1016/j.ajem.2017.08.049 28855065

[5] Porto BM. Improving triage performance in emergency departments using machine learning and natural language processing: a systematic review. BMC Emerg Med. 2024;24(1). 10.1186/s12873-024-01135-2. 10.1186/s12873-024-01135-2 PMC11575054 39558255

[6] Kuo K-M, Wu W-S, Chang CS. A meta-analysis of the diagnostic test accuracy of artificial intelligence for predicting emergency department revisits. J Med Syst. 2025;49(1). 10.1007/s10916-025-02210-2. 10.1007/s10916-025-02210-2 40522351

[7] Lee Y-C, Ng C-J, Hsu C-C, Cheng C-W, Chen S-Y. Machine learning models for predicting unscheduled return visits to an emergency department: a scoping review. BMC Emerg Med. 2024;24(1). 10.1186/s12873-024-00939-6. 10.1186/s12873-024-00939-6 PMC10826225 38287243

[8] Chen T, Guestrin C. Xgboost: a scalable tree boosting system. Proceedings of the 22nd ACM SIGKDD International Conference on Knowledge Discovery and Data Mining. KDD’16. New York, NY, USA: ACM; 2016, pp. 785–94). 10.1145/2939672.2939785.

[9] Lin Y-W, Zhou Y, Faghri F, Shaw MJ, Campbell RH. Analysis and prediction of unplanned intensive care unit readmission using recurrent neural networks with long short-term memory. PLoS One. 2019;14(7):0218942. 10.1371/journal.pone.0218942. 10.1371/journal.pone.0218942 PMC6613707 31283759

[10] Zebin T, Chaussalet TJ. Design and implementation of a deep recurrent model for prediction of readmission in urgent care using electronic health records. 2019 IEEE Conference on Computational Intelligence in Bioinformatics and Computational Biology (CIBCB). Siena, Italy: IEEE; 2019, pp. 1–5). doi: 10.1109/CIBCB.2019.8791466.

[11] Porto BM, Fogliatto FS. Enhanced forecasting of emergency department patient arrivals using feature engineering approach and machine learning. BMC Med Inf Decis Mak. 2024;24(1). 10.1186/s12911-024-02788-6. 10.1186/s12911-024-02788-6 PMC11653554 39696224

[12] Sung C-W, Ho J, Fan C-Y, Chen C-Y, Chen C-H, Lin S-Y, Chang J-H, Chen J-W, Huang EP-C. Prediction of high-risk emergency department revisits from a machine-learning algorithm: a proof-of-concept study. BMJ Health Care Inf. 2024;31(1):100859. 10.1136/bmjhci-2023-100859. 10.1136/bmjhci-2023-100859 PMC11043771 38649237

[13] Kim M, Lee YS, Park Y, Jung A, So H, Park J, Park J, Choi D, Kim S, Park S. Deep learning for predicting rehospitalization in acute heart failure: Model foundation and external validation. ESC Heart Fail. 2024;11(6):3702–12. 10.1002/ehf2.14918. 38981003 10.1002/ehf2.14918 PMC11631275

[14] Lopez K, Li H, Lipkin-Moore Z, Kay S, Rajeevan H, Davis JL, Wilson FP, Rochester CL, Gomez JL. Deep learning prediction of hospital readmissions for asthma and copd. Respir Res. 2023;24(1). 10.1186/s12931-023-02628-7. 10.1186/s12931-023-02628-7 PMC10720134 38093373

[15] Ashfaq A, Sant’anna A, Lingman M, Nowaczyk S. Readmission prediction using deep learning on electronic health records. J Biomed Inf. 2019;97:103256. 10.1016/j.jbi.2019.103256. 10.1016/j.jbi.2019.103256 31351136

[16] Tang S, Tariq A, Dunnmon JA, Sharma U, Elugunti P, Rubin DL, Patel BN, Banerjee I. Predicting 30-day all-cause hospital readmission using multimodal spatiotemporal graph neural networks. IEEE J Biomed Health Inform 2023, 1–12) doi: 10.1109/jbhi.2023.3236888. 10.1109/JBHI.2023.3236888 PMC11073780 37018684

[17] Iwana BK, Uchida S. An empirical survey of data augmentation for time series classification with neural networks. PLoS One. 2021;16(7):0254841. 10.1371/journal.pone.0254841. 10.1371/journal.pone.0254841 PMC8282049 34264999

[18] Tang Q, Cen X, Pan C. Explainable and efficient deep early warning system for cardiac arrest prediction from electronic health records. Math Biosci And Eng. 2022;19(10):9825–41. 10.3934/mbe.2022457. 10.3934/mbe.2022457 36031970

[19] Kazijevs M, Samad MD. Deep imputation of missing values in time series health data: a review with benchmarking. J Biomed Inf. 2023;144:104440. 10.1016/j.jbi.2023.104440. 10.1016/j.jbi.2023.104440 PMC10529422 37429511

[20] Bai S, Kolter JZ, Koltun V. An empirical evaluation of generic Convolutional and recurrent networks for sequence modeling. arXiv. 2018. 10.48550/ARXIV.1803.01271.

[21] Wang J-Y, Hsu S-Y, Sun J-T, Ko C-H, Huang C-H, Tsai C-L, Fu L-C. Internal and external validation of a deep learning-based early warning system of cardiac arrest with variable-length and irregularly measured time series data. J Healthcare Inf Res. 2025. 10.1007/s41666-025-00188-7.

[22] Namgung M, Lee DH, Bae SJ, Chung HS, Kim K, Lee CA, Kim DH, Kim EC, Lim JY, Han SS, Choi YH. The impact of covid-19 pandemic on revisits to emergency department. Australas Emerg Care. 2023;26(3):221–29. 10.1016/j.auec.2023.01.002. 10.1016/j.auec.2023.01.002 PMC9874043 36717326

[23] Hond AAH, Shah VB, Kant IMJ, Van Calster B, Steyerberg EW, Hernandez-Boussard T. Perspectives on validation of clinical predictive algorithms. npj Digit Med **6**(1). 2023. 10.1038/s41746-023-00832-9. 10.1038/s41746-023-00832-9 PMC10163568 37149704

[24] Moons KGM, Altman DG, Reitsma JB, Ioannidis JPA, Macaskill P, Steyerberg EW, Vickers AJ, Ransohoff DF, Collins GS. Transparent reporting of a multivariable prediction model for individual prognosis or diagnosis (tripod): explanation and elaboration. Ann Intern Med. 2015;162(1):1–73. 10.7326/m14-0698. 10.7326/M14-0698 25560730

[25] Ismail F, A. Benchmarking deep learning interpretability in time series classification. Proceedings of the 26th ACM SIGKDD International Conference on Knowledge Discovery & Data Mining. 2020, pp. 2633–43.

